# Stacking models of brain dynamics to improve prediction of subject traits in fMRI

**DOI:** 10.1162/imag_a_00267

**Published:** 2024-08-20

**Authors:** Ben Griffin, Christine Ahrends, Chetan Gohil, Fidel Alfaro-Almagro, Mark W. Woolrich, Stephen M. Smith, Diego Vidaurre

**Affiliations:** Oxford Centre for Functional MRI of the Brain (FMRIB), Wellcome Centre for Integrative Neuroimaging, Nuffield Department of Clinical Neurosciences, University of Oxford, Oxford, United Kingdom; Center of Functionally Integrative Neuroscience, Department of Clinical Medicine, Aarhus University, Aarhus, Denmark; Oxford Centre for Human Brain Activity (OHBA), Wellcome Centre for Integrative Neuroimaging, Department of Psychiatry, University of Oxford, Oxford, United Kingdom

**Keywords:** fMRI, prediction, brain dynamics, model combination, hidden Markov model, subject differences

## Abstract

Beyond structural and time-averaged functional connectivity brain measures, modelling the way brain activity dynamically unfolds can add important information to our understanding and characterisation of individual cognitive traits. One approach to leveraging this information is to extract features from models of brain network dynamics to predict individual traits. However, these predictions are susceptible to variability due to factors such as variation in model estimation induced by the choice of hyperparameters. We suggest that, rather than merely being statistical noise, this variability may be useful in providing complementary information that can be leveraged to improve prediction accuracy. To leverage this variability, we propose the use of stacking, a prediction-driven approach for model selection. Specifically, we combine predictions developed from multiple hidden Markov models—a probabilistic generative model of network dynamics that identifies recurring patterns of brain activity—to demonstrate that stacking can slightly improve the accuracy and robustness of cognitive trait predictions. By comparing analysis from the Human Connectome Project and UK Biobank datasets, we show that stacking is relatively effective at improving prediction accuracy and robustness when there are enough subjects, and that the effectiveness of combining predictions from static and dynamic functional connectivity approaches depends on the length of scan per subject. We also show that the effectiveness of stacking predictions is driven by the accuracy and diversity in the underlying model estimations.

## 1 Introduction

A major objective in neuroscience is to develop predictive models that aim to discover associations between brain data and subject traits (e.g., clinical or behavioural measures). Accurate and robust predictions are essential if models are to be used for real-world applications, particularly in clinical settings (Dinsdale et al., 2022; Kaufmann et al., 2019; Mourao-Miranda et al., 2012; Shahab et al., 2019). In this paper, our objective is to develop predictive models using resting-state functional magnetic resonance imaging (rfMRI), where typically functional connectivity (FC) between various brain regions is estimated by averaging data across several minutes. Prior research has revealed associations between this static (or time-averaged) FC and working memory performance (Hampson et al., 2006), higher-level cognitive processing (Hasson et al., 2009), and various other behavioural phenotypes (Smith et al., 2015).

However, a time-averaged approach overlooks the dynamic fluctuations that may occur during a brain scan—referred to as time-varying (dynamic) FC approaches. There is some evidence highlighting the behavioural relevance of the dynamics of the functional connectome beyond what is captured by static FC alone. For instance, in comparison with static FC, studies have found stronger associations between time-varying FC and intelligence (Vidaurre et al., 2021), as well as various personality traits (Jia et al., 2014). Additionally, research has shown that combining dynamic and static FC yields superior predictive accuracy for predicting attention task performance in comparison with models driven solely by static FC (Fong et al., 2019; Kavitha et al., 2019; Pievani et al., 2011).

A common approach to examining brain dynamics is the sliding time window approach (Sakoğlu et al., 2010). Although there are several variants of this approach (Allen et al., 2014; Leonardi & Van De Ville, 2015; Liégeois et al., 2016), the estimation of FC is inherently noisy and can be influenced by factors such as length and placement of the time windows, affecting the model’s ability to detect temporal variations of interest (Mokhtari et al., 2019; Preti et al., 2017). An alternative approach to examining brain dynamics is to use generative models of brain network dynamics that are trained on the entire data set, and then extract features that can predict individual subject traits. One example of this is the use of multivariate autoregressive models, which can capture dynamic FC at a temporal resolution of a few seconds and have been used to predict task-based phenotypes better than static FC (Liégeois et al., 2019).

In our study, we employed the hidden Markov model (HMM), a probabilistic model of temporal dynamics of amplitude and FC that characterises the data using a discrete number of amplitude and FC states and transitions between them (Vidaurre et al., 2017). By modelling the brain data using different parameterisations of the HMM, we aim to discover distinct and complementary representations of the data. As previously demonstrated, the HMM can be combined with machine learning methods to predict subject traits from fMRI recordings (Ahrends et al., 2024; Vidaurre et al., 2021). Nevertheless, the optimal selection and utilisation of HMM features to maximise prediction performance remain unclear. In this paper, we investigate various approaches to generate features for improving prediction performance. We then harness the computational power of kernel methods (Schölkopf & Smola, 2002) to predict subject-specific phenotypes from these features.

Regardless of the chosen generative model, there can be two potential sources of variation in these predictions. First, there can be run-to-run variability (e.g., due to different initialisations of model parameters or the stochasticity in parameter updates); the HMM inference is an example of this (Alonso & Vidaurre, 2023), which can impact subsequent predictions made using features derived from the fitted HMM. Second, the choice of generative model hyperparameters, such as the model order (i.e., number of HMM states) or the strength of regularisation, affects the result of the estimation. Modifying these hyperparameters can add an additional factor of variability. While this variability may be detrimental if driven by estimation noise, it can also represent the discovery of distinct and complementary information (across different analyses of the same data).

To address these challenges, we employed a stacked generalisation framework (Breiman, 1996; Wolpert, 1992) that aimed to reduce the variability driven by estimation noise to improve robustness, while leveraging the useful variability to improve the accuracy in predicting subject traits. Previous studies have used stacking to integrate multimodal neuroimaging data for age prediction (Engemann et al., 2020) and mild cognitive impairment classification (Vaghari et al., 2022). Here, we used stacking to substitute model selection with model integration—in other words, our approach bypassed the complex decision of selecting the best HMM hyperparameter choices, albeit at the expense of increased computation. Specifically, our method aimed to integrate complementary descriptions of brain dynamics, such as capturing different temporal scales, as they are captured by different configurations of the HMM (i.e., across a wide range of hyperparameters), to produce a single, superior model.

Focusing on rfMRI due to its wide availability, we explore the efficacy of our stacking framework on data from two large-scale neuroimaging datasets: UK Biobank (UKB) (Sudlow et al., 2015) and the Human Connectome Project (HCP) (Van Essen et al., 2013). For both datasets, we explored the relationships between rfMRI data and a range of cognitive traits. By doing so, we found that stacking predictions generated from different configurations of the HMM frequently outperformed simpler approaches in terms of robustness and accuracy.

## 2 Materials and Methods

To predict a set of cognitive traits from models of rfMRI FC dynamics, our approach involved multiple steps. In brief, we first independently ran the HMM on the rfMRI data multiple times, generating a prediction from each HMM (referred to as base-level predictions). Subsequently, we used these base-level predictions as input features for a meta-model to generate a stacked prediction.

**Fig. 1.**
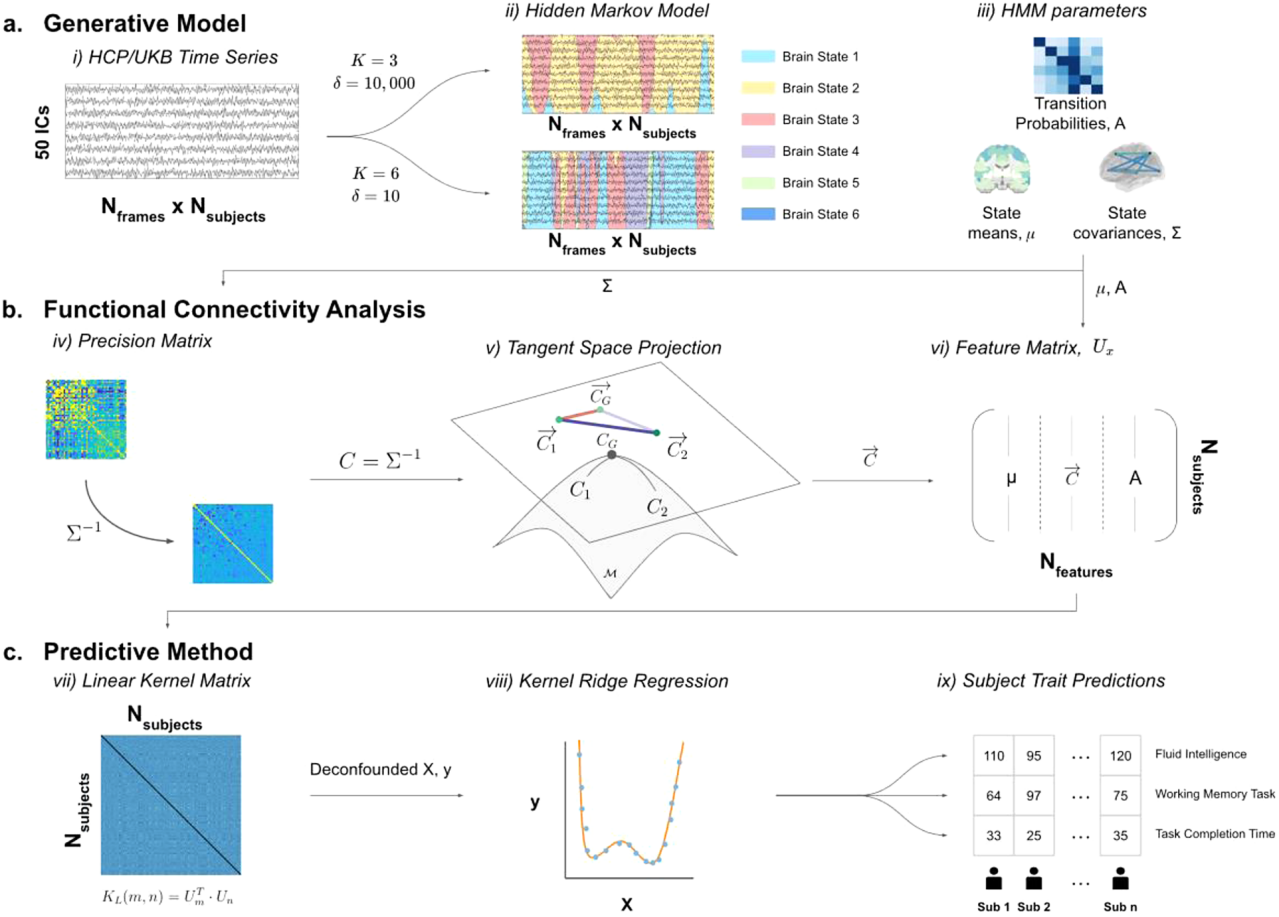
Procedure for predicting subject traits from resting-state fMRI (rfMRI) timeseries. (a) Generative Model. (i) rfMRI in group-ICA parcellations with 50 ICs is concatenated across all subjects from UKB and HCP (separately). (ii), (iii) The hidden Markov model is trained on the timeseries, where different HMM hyperparameters, such as number of states (K) or a parameter controlling the prior probability of remaining in the same state (δ), lead to different HMM descriptions. (b) Functional Connectivity Analysis. (iv) Regularised precision matrices are estimated by finding the inverse of the covariance matrix for each brain state. (v) The precision matrices are projected onto the tangent space to improve the validity of then applying Euclidean methods. (vi) The elements of the tangent space projection are vectorised for each subject and combined with the brain state means and transition probabilities to form the feature matrix. (c) Predictive Method. (vii) A linear kernel matrix is generated from the features. (viii) This kernel is used as a predictor in a (nested) cross-validated, de-confounded kernel ridge regression model to predict subject traits, where the optimal regularisation parameters are found via 10-fold cross-validation. (ix) This last step is carried out independently to predict multiple cognitive traits.

The process used to produce a base-level prediction is outlined in Figure 1. For each base-level prediction, an HMM was run on a temporally concatenated fMRI timeseries of all subjects to obtain a group-level HMM (Vidaurre et al., 2017, 2021), from which subject-specific HMM parameters were inferred using dual estimation (Vidaurre et al., 2021) (Fig. 1a). We then turn the covariance matrices of each brain state into a precision matrix, before projecting them onto the (Riemannian manifold) tangent space. The elements of these projected matrices are then vectorised for all subjects to develop a feature matrix used for prediction (Fig. 1b). Finally, subject-specific trait predictions were generated using a kernel ridge regression (KRR) model with a linear kernel (Fig. 1c).

Specifically, we generated 100 base-level predictions in this way, independently estimating an HMM for each base-level prediction from the same fMRI timeseries: 50 times with fixed hyperparameters (to investigate the run-to-run variability of the HMM) and 50 times with varying hyperparameters (to investigate variability induced by varying the hyperparameters of the HMM). We then generated two stacked predictions by separately combining the two sets of 50 predictions. We now present all the steps that integrate this procedure in more detail and introduce the two datasets to which we applied the framework, UKB and HCP.

For completeness, we also compared this approach with the Fisher kernel method (see Supplementary Methods 1.1), a mathematically principled approach to predicting target variables from an HMM.

### 2.1 HCP neuroimaging and non-imaging data

We used publicly available rfMRI data from 1,001 subjects from the HCP dataset (Van Essen et al., 2013). The full acquisition details and preprocessing pipeline for the HCP dataset are described in Van Essen et al. (2012). In brief, 3T whole-brain fMRI data were acquired with a spatial resolution of 2 × 2 × 2 mm and a temporal resolution of 0.72 seconds. The preprocessing pipeline is described in Smith et al. (2013), but the primary steps included motion correction, high-pass temporal filtering to remove the linear trends of the data, and artefact removal using ICA+FIX (Griffanti et al., 2014). A parcellation of 50 independent components (ICs) was obtained by performing MELODIC (Beckmann & Smith, 2004). We chose an ICA-based parcellation as they are more reliable than other functional and anatomical parcellations in detecting temporal changes in FC (Ahrends et al., 2022). Additionally, ICA-based parcellations outperformed alternative parcellations (e.g., Shen, Yeo) in terms of predictive power when applied to fMRI data from UKB and HCP (Ahrends et al., 2022; Pervaiz et al., 2020). For each participant, this resulted in 50 timeseries, composed of 4,800 time points across 4 scanning sessions (with 1,200 time points in each session) of approximately 15 minutes each. For performance evaluation, we selected all traits related to fluid intelligence, as well as the traits given by the unadjusted cognitive test scores. This resulted in 15 cognitive traits. The list of these 15 traits can be found in Supplementary Table SI-1.

As in Vidaurre et al. (2017), we controlled for sex and motion. Confounds were regressed out from both the predictor and target variables using linear regression. Specifically, confound regression parameters were estimated from the training set and subsequently applied to both the training and testing data. Additionally, we accounted for familial relationships when assigning subjects to cross-validation folds, to make sure related subjects were never split across folds.

### 2.2 UKB neuroimaging and non-imaging data

In addition to the HCP dataset, we tested our models on 16,352 subjects from the UKB dataset (Sudlow et al., 2015). The full acquisition details and preprocessing pipeline for the UKB dataset are described in Smith et al. (2022). In brief, 3T fMRI data were acquired for each participant, consisting of 490 time points per session at a TR of 0.735 seconds and with 2.4 mm spatial resolution. Preprocessing was performed using the standard UKB pipeline, which is similar to the HCP pipeline, including brain extraction, motion correction, structured artefact removal using ICA+FIX (Griffanti et al., 2014), high-pass temporal filtering, and registration to MNI152 space (Alfaro-Almagro et al., 2018). To allow for a more direct comparison between UKB and HCP, we used surface-based node timeseries generated using 50-dimensional group-ICA surface maps from the HCP S1200 dataset (Van Essen et al., 2013). This resulted in 50 timeseries, composed of 490 time points across a 6-minute scanning session for each patient.

To allow for a more direct comparison between datasets, we matched the number of traits investigated in UKB with the number investigated in HCP. We first excluded cognitive traits with missing recorded values for over half of UKB subjects, before performing preliminary analyses to generate predictions for the remaining 450 cognitive traits in UKB (of the 1,331 cognitive traits in total), from which we selected the top-performing 15 traits in terms of prediction accuracy. The rationale behind selecting the traits which could be predicted most accurately was to demonstrate the potential of stacking compared with alternative approaches. Since stacking involves combining predictions from base estimators, these underlying estimators must exhibit a certain level of accuracy for stacking to be useful.

When choosing the traits to predict, it was important to ensure that the stacking process remained unbiased towards any particular HMM configuration, or towards our stacked approach, given that the primary focus of our investigation was to investigate the stacking of predictions from dynamic FC. Additionally, in our comparison of predictions obtained from dynamic FC versus static FC, we aimed to avoid bias towards any dynamic approach. To achieve this, we initially conducted preliminary analysis to select traits with the highest predictive accuracy using predictions from static FC. The prediction accuracies for all 450 cognitive traits are provided in Supplementary Figure SI-1 and the list of the selected 15 traits can be found in Supplementary Table SI-2.

As with HCP, confounds were regressed out from both the predictor and target variables; the regression parameters were estimated from the training set and applied to both the training and test set. We employed a reduced set of confounds extracted from a comprehensive set of 602 UKB imaging confounds provided by Alfaro-Almagro et al. (2021). To generate our reduced set of confounds, we first selected a subset of conventional confounds including sex, scanning site, head size, and head motion (which use FSL’s FEAT (Woolrich et al., 2001) and EDDY (Andersson et al., 2016, 2017)). Then, we reduced the remaining confounds by using the singular value decomposition and selecting the top principal components which accounted for 85% of the variance.

### 2.3 The hidden Markov model

The hidden Markov model is a generative probabilistic model that can find recurrent patterns of amplitude and FC from fMRI (Vidaurre et al., 2017). The model assumes that there is a discrete number of hidden states, each associated with a unique probabilistic model, that generates the observed data. In the main body of this paper, we chose to model both the FC and signed amplitude in the observation model for each state. However, it is possible to parametrise the HMM to focus primarily on FC, where each hidden state is characterised by a Gaussian distribution observation model with no mean vector parameter (which corresponds to the signed amplitude) and a full covariance matrix to capture the pairwise covariance across regions (or equivalently a Wishart distribution; Vidaurre et al., 2021). We have also considered this alternative parametrisation (see Supplementary Fig. SI-2).

An adequate choice for fMRI is to model these brain states as Gaussian distributions, where each state k (of a total K states) is governed by two sets of parameters—a mean vector $\mu_{k}$, which can be interpreted as the mean amplitude for each of M brain regions from a specified parcellation (here *M* group-ICA components), and a covariance matrix $\Sigma_{\text{k}}$, which can be interpreted as FC between the M brain regions. The model inference also estimates the initial probabilities that the timeseries start in each of the K states, given by π, as well as the probabilities of transitioning between states, given by a transition probability matrix A.

The HMM used to represent the fMRI data is described by this set of parameters, θ (see Fig. 1a), where μ and Σ represent the mean vectors and covariance matrices across all states:

$$\theta =\left[\pi ,A,\mu ,\Sigma \right]\;\pi \in {\mathbb{R}}^{1xK},\;A\in {\mathbb{R}}^{KxK},\;\;\mu \in {\mathbb{R}}^{KxM},\;\Sigma \in {\mathbb{R}}^{KxMxM}.$$ (1)

The optimal parameters are estimated from the data at the group level (i.e., the state probability distributions are the same for all subjects)^*^. Following this, subject-specific HMM parameters can be determined using dual estimation (Vidaurre et al., 2021), which can subsequently be used as features for prediction.

In the HMM generative process, we assume that each time step t of the observed fMRI timeseries $X_{t}$ involves sampling from a Gaussian distribution with mean $\mu_{k}$ and covariance $\Sigma_{k}$ when state k is active:

$$X_{t}|q_{t}\;=k\;\sim \;N\left(\mu_{k},\;\Sigma_{k}\right),$$ (2)

where $q_{t}$ is the active state at time t. This currently active state, $q_{t}$, depends on the previous state $q_{t-1}$ and is determined by the transition probabilities A. Consequently, the sequence of states is generated by sampling from a categorical distribution with parameters:

$$q_{t}\text{|}(q_{t-1}=k\text{)}\sim \;Cat(A_{k}),$$ (3)

where $A_{k}$ represents the k-th row of the transition probability matrix.

When applying the HMM, in this study there were two potential sources of variability. The first we refer to as run-to-run estimation variability, which can arise both from the random initialisation of the HMM estimation process for a given dataset and from the stochastic inference method applied. Random initialisation refers to the random assignment of parameter values, such as the state parameters or transition probabilities, at the beginning of the training process. Furthermore, we used an efficient stochastic approach for the HMM estimation, which applies the principles of stochastic optimisation to variational inference (Vidaurre et al., 2018). This approach is computationally cheaper and suitable for large neuroimaging datasets such as UKB and HCP. However, it introduces variability in the estimation of the state-time courses, which are determined by the Baum-Welch algorithm (Baum et al., 1970). At each iteration, the estimation of the state-time course is updated based on a random subset of subjects, which is a noisy but computationally efficient approach. Since the HMM optimisation can converge to local minima, different initialisations and batches of subjects for the estimation can impact the accuracy of subsequent predictions.

The second source of variability comes from the selection of the hyperparameters of the HMM, which are set by the user. We refer to this as hyperparameter selection variability. By varying the hyperparameters, we can discover distinct patterns of FC, for example, across different time scales (Ahrends et al., 2022). In our study, we focused on varying two HMM hyperparameters: the number of states (K) and the prior probability of remaining in the same state (δ), which is parametrised by the prior Dirichlet distribution concentration parameter of the corresponding prior distribution^†^ (Masaracchia et al., 2023), which effectively influences the time scale of the estimate. This parameter determines the size of the diagonal elements in the prior distribution of the hidden states (i.e., the transition probability matrix).

In total, we fit the HMM to the same rfMRI timeseries 100 times: 50 times with fixed hyperparameters^‡^ (i.e., with random initialisations and random batches of subjects for the stochastic inference) and 50 times with varying hyperparameters^§^ (i.e., with additional variability coming from the selection of hyperparameters). When fixing the hyperparameters, we chose δ=10 , the default setting in the HMM-MAR toolbox, and K=6 , commonly used values in recent literature (Alonso & Vidaurre, 2023; Quinn et al., 2018; Vidaurre et al., 2018). The choice of states represents a compromise between having a higher number of states, which can increase the chances of certain states being present in only a subset of subjects (Ahrends et al., 2024), and a lower number of states, which can increase the chance of assigning entire sessions to a single state (i.e., model stasis) (Ahrends et al., 2022). Nevertheless, the specific choice of hyperparameters is less critical to our study, as our focus when fixing the hyperparameter is to investigate run-to-run variability which could be done with an alternative configuration.

When we varied the hyperparameters, our objective was to investigate both run-to-run variability and hyperparameter selection variability, irrespective of the specific hyperparameter settings employed. Therefore, we used a wide range of hyperparameters and repeated each hyperparameter combination twice with different initialisations and different subject batches for the stochastic inference. As a result, we anticipated a greater degree of variability when adjusting the hyperparameters of the HMM, since we expect to encounter both types of variability.

#### 2.3.1 Generating features for prediction from an HMM estimate

After performing dual estimation, subject-specific HMM features (including the brain state covariances and means, and transition probability matrices) can be used for prediction. However, using full covariance matrices to estimate functional connectivity does not differentiate between direct and indirect connections between brain regions (i.e., connections mediated through other brain regions). Partial correlations can address this limitation, potentially leading to superior predictions (Pervaiz et al., 2020). Partial correlation between two brain regions is defined as the correlation between them after controlling for all other regions.

In the context of dynamic FC, we can calculate the partial correlation matrix from the covariance matrix of a given brain state, $\Sigma_{\text{k}}$. To determine the partial correlation matrix, we can use the precision matrix (i.e., the inverse of the covariance matrix). Specifically, given the elements of the precision matrix, $\Sigma_{\text{k}}^{-1}=\Omega_{\text{k}}=p_{ij}$ , partial correlations for the corresponding brain state, $\rho_{k}$, are given by

$$\rho_{k}\;=\;-\frac{p_{ij}}{\sqrt{p_{ii}p_{jj}}}.$$ (4)

To obtain the precision matrix, we can simply use the Cholesky decomposition (Krishnamoorthy & Menon, 2013) to factorise the covariance matrix into an upper triangular matrix, R,

$$\Sigma_{\text{k}}=R^{T}R,$$ (5)

before taking the inverse:

$$\Omega_{k}\;=\;\Sigma_{\text{k}}^{-1}=R^{-1}{\left(R^{-1}\right)}^{T}.$$ (6)

We performed this analysis for each brain state for a given HMM, and subsequently for all HMMs.

To mitigate the issue of instability which can arise from inverting the covariance matrix, we incorporate Tikhonov regularisation (Golub et al., 1999), also known as L2-regularisation. Specifically, we add a penalty term to the covariance matrix before inverting it to calculate the regularised precision matrix for a given state, ${\bar{\Omega }}_{k}$:

$${\bar{\Omega }}_{k}\;={\left(\Sigma_{\text{k}}\;+aI\right)}^{-1},$$ (7)

where a is the regularisation parameter and I the identity matrix.

We found that the optimal level of regularisation depended on the number of HMM states (see Supplementary Methods 1.2 for details).

Both the set of covariance matrices and precision matrices corresponding to a given brain state are inherently symmetric and positive definite. The space of symmetric positive definite matrices forms a Riemannian manifold (Jayasumana et al., 2013), for which Euclidean metrics are not suitable. Therefore, we instead use a method to approximate the Riemannian distance between covariance matrices, which involves projecting the matrices onto the tangent space.

To carry out this projection, it is necessary to select a reference point for the tangent space placement, ideally situated close to the subjects’ projected covariance matrix. Typically, an average of all subjects’ covariance matrices is chosen as the reference point, for which we here use the Riemannian average (Moakher, 2005). After calculating the group average for the reference point, $C_{G}$, we use the logarithmic map (Barachant et al., 2013) to project a covariance matrix for a given subject, $C_{n\;}$ , onto the tangent space (see Fig. 1b):

$$\vec{C_{n}\;}=\;\log_{m}\left(C_{G}^{-\frac{1}{2}}\;C_{n}C_{G}^{-\frac{1}{2}}\right).$$ (8)

This can be done for both the set of covariance matrices and the precision matrices. Subsequently, the features of these projected matrices can be used as features for prediction, alongside other relevant HMM features such as the state means and transition probabilities.

### 2.4 Static FC

To compare the performance of our dynamic FC predictions with simpler methods that do not consider brain dynamics, we also used time-averaged FC (also referred to as static FC) for prediction. To be comparable with the dynamic FC predictions, we repeated the same analysis pipeline as for the covariance matrices from dynamic FC. We first computed the covariance among all pairs of brain regions across the entire timeseries. Subsequently, we calculated the precision matrix by inverting the covariance matrix with L2-regularisation. Finally, we projected the set of precision matrices onto the tangent space, before using these projected matrices as features for prediction using our chosen kernel-based prediction approach.

### 2.5 Kernel ridge regression (KRR)

In this study, we adopted a kernel-based approach to predicting behavioural traits from HMM features, using a simple linear kernel. For subjects m and n with feature vectors $U_{m}$ and $U_{n},$ respectively, elements of the linear kernel matrix, $K_{L}$, were calculated via:

$$K_{L}(m,n)=U_{m}\;T\cdot U_{n}.$$ (9)

The dot product was computed for all subject pairs to obtain the similarity matrix, which could then be used with any suitable kernel prediction method or classifier to determine the relationship between the subjects’ time-varying activity and FC patterns and their cognitive traits.

In this study, we used KRR due to its prevalence in neuroimaging literature, efficiency, and ability to achieve comparable performance with more complex approaches such as deep learning (T. He et al., 2020; Mihalik et al., 2019).

KRR is the kernelised version of ridge regression (Saunders et al., 1998). Given a subject trait to predict, y, regression coefficients, α, are determined by solving the optimisation problem:

$$\underset{\alpha }{\min}{\left|\left|K_{L}\alpha -y\right|\right|}^{2}\;\text{subject to}\;{\left|\left|\alpha \right|\right|}^{2}=\lambda ,$$ (10)

where λ is the L2-regularisation chosen by cross-validation. We employed a nested cross-validation scheme to identify the optimal level of regularisation across 10 distinct folds, which is important to reduce the risk of overfitting.

The optimal regression coefficients, $\alpha^{*}$, can then be estimated as

$$\alpha^{*}\;={\left(K_{L}+\lambda I\right)}^{-1}y,$$ (11)

where I is the identity matrix.

We used KRR to generate base-level predictions corresponding to independent runs of the HMM on the same data, for 15 cognitive traits from the UKB and HCP datasets.

### 2.6 Stacking

Stacking, or stacked generalisations, is a method which can be employed to potentially improve the predictions generated from methods such as KRR. Specifically, stacking is a technique that combines the predictions from multiple base models by training a meta-model that uses the base model predictions as input features.

Given multiple base models, the objective is to determine optimal model coefficients, or stacking weights, for combining the base-level predictions to generate the best stacked predictions for a given subject trait. To achieve this, a common approach is to use the base-level predictions as input features in a constrained linear regression, where the model coefficients are forced to be non-negative and sum to 1 (Breiman, 1996):

$$\underset{\beta }{\min}{\left|\left|\hat{Y}\beta -y\right|\right|}^{2}\;\;\text{subject to}\;\sum_{i=1}^{N}\beta_{i}\;=1,\;\beta_{i}\;\geq \;0\;\forall i=1\;,\ldots ,N$$ (12)

where $\hat{Y}$ is the feature matrix (formed of base-level predictions), y represents the corresponding subject traits, and β are the stacking weights to be determined.

The base-level predictions are likely to be highly correlated, particularly when generated from HMMs with identical hyperparameters. Therefore, if unconstrained least squares is used to determine the stacking weights, there is no guarantee that the resulting stacked prediction would stay within the range of the base-level predictions and generalisation may be poor; however, by imposing non-negativity and sum-to-1 constraints, an interpolating prediction is developed (see Breiman (1996) for details).

The primary contribution of this paper is the implementation of a stacked generalisation scheme (Wolpert, 1992) to combine predictions obtained from multiple HMM runs using the previously described approaches. After developing multiple base-level predictions, we used a two-layered nested cross-validation scheme to generate out-of-sample stacked predictions (i.e., predictions for unseen subjects).

**Fig. 2.**
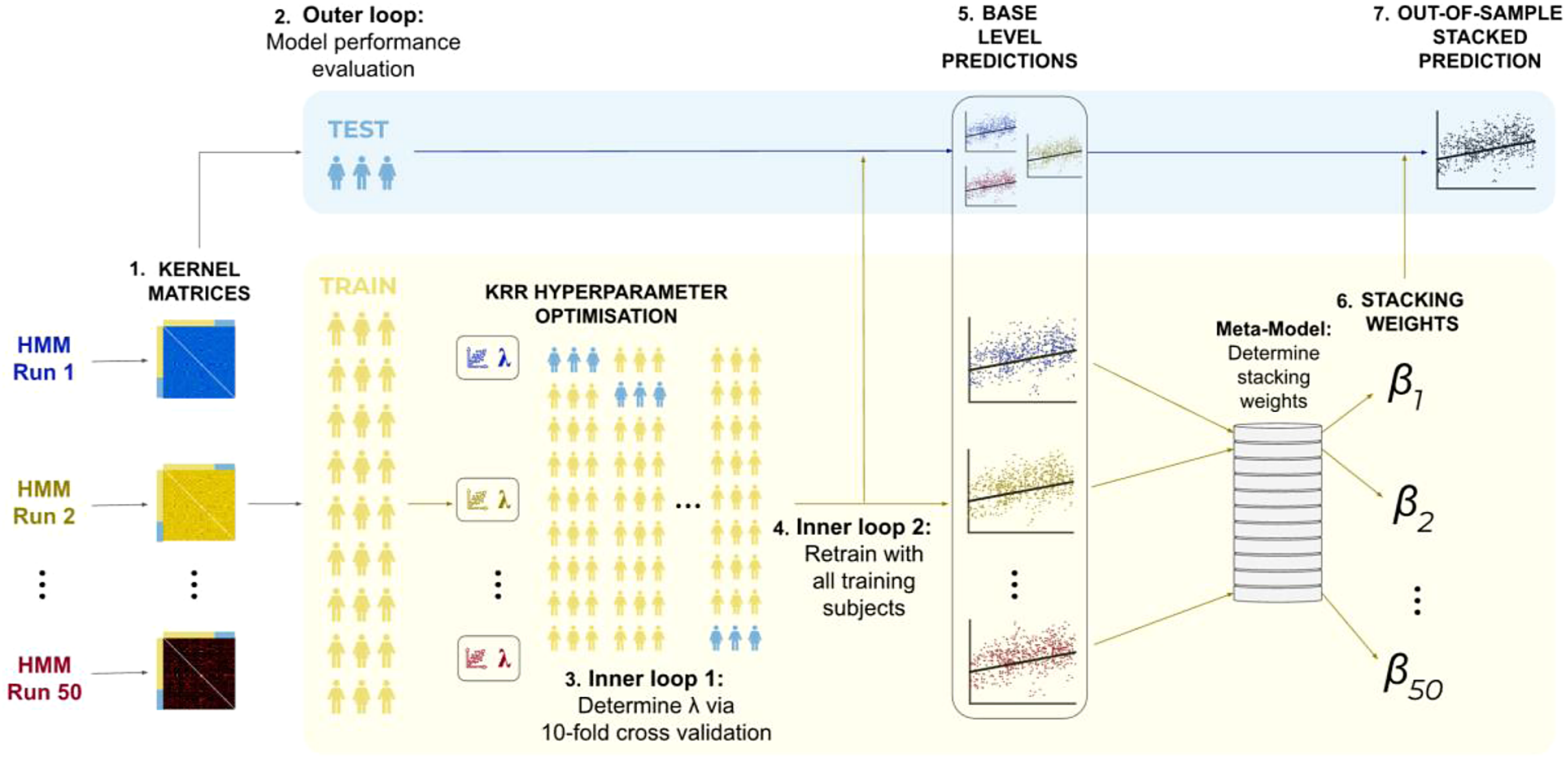
Cross-validation framework for stacking 50 base-level predictions developed from kernel matrices generated from the features of 50 distinct hidden Markov models. (1) Kernel matrices: 50 group-level HMMs were run on the concatenated rfMRI timeseries across subjects, from which subjects-by-subjects kernel matrices were developed from the features of each HMM. (2) Outer loop: the subjects were split into 10 cross-validation folds, where each fold was selected in turn as an outer loop test set (used for stacked model performance evaluation) and the remaining 9 folds are used as an outer loop training set. The training subjects were further divided into 10 cross-validation folds, which are used for 2 purposes. (3) Inner loop 1: this loop was used to optimise the L2-regularisation parameter, λ. (4) Inner loop 2: using the optimised λ from inner loop 1, base-level predictions were developed for all inner loop subjects (or equivalently the outer loop training subjects). (5) Base-level predictions: Separately, base-level predictions are generated for the outer loop test subjects using the optimised hyperparameter from inner loop 1. (6) Stacking weights: The 50 base-level estimators from inner loop 2 were then stacked, with the stacking weights constrained to be non-negative and sum to 1. (7) Out-of-sample stacked prediction: finally, the stacking weights are combined with the base-level predictions for the outer loop test subjects to produce out-of-sample stacked predictions for a selected cognitive trait. This is repeated for all 10 outer loop cross-validation folds.

A summary of the stacking and nested cross-validation framework is depicted in Figure 2. Initially, the subjects were divided into 10 folds. Each fold was sequentially chosen as the outer loop test set, while the remaining nine folds formed the outer loop training set. This outer loop served as the first layer of the nested cross-validation and was used to evaluate the performance of the stacked model. The subjects in the outer loop training set (i.e., 90% of the full sample) were further divided into 10 folds, which served as the 9 training folds and 1 test fold for the inner loops of the cross-validation. This constituted the second layer of the nested cross-validation, involving two inner loops using the same cross-validation folds. These inner loops served two purposes: optimising the regularisation parameter and determining stacking weights.

The first inner cross-validation loop (see *inner loop 1* in Fig. 2) was used to tune the L2-regularisation parameters of the KRR models. This process was carried out separately for each kernel matrix obtained from the respective HMM. Following that, the second inner cross-validation loop (see *inner loop 2* in Fig. 2) was used to determine stacking weights. In this step, the optimised KRR regularisation parameters (from inner loop 1) were used to generate 50 base-level predictions for all inner loop subjects (or, equivalently, the outer loop training subjects; see *inner loop 2* in Fig. 2). These base-level predictions were subsequently used as input features in a meta-model to generate stacking weights, where the stacking weights were forced to be non-negative and sum to 1.

Finally, we developed stacked predictions for the outer loop test set. To do this, we used the optimised KRR regularisation parameters generated in inner loop 1 to generate base-level predictions for the outer loop test set. We then combined these outer loop test set predictions with the stacking weights generated from inner loop 2 (in other words the outer loop training set) to produce out-of-sample test set stacked predictions, used to evaluate model performance. We completed this analysis for 50 HMMs with fixed hyperparameter (to test run-to-run variability of the HMM) and 50 HMMs with varying hyperparameters (to test hyperparameter selection variability). This resulted in 2 sets of 50 base-level predictions and 2 corresponding stacked predictions. Through this approach, we sought to create a more robust stacked prediction with superior accuracy.

By varying the hyperparameters of the HMM, our objective was to explore different configurations that could reveal unique brain state distributions, accurately capturing information across subjects. However, adjusting these hyperparameters can sometimes lead to inadequate characterisation of brain data. For instance, the choice of the number of states can impact model stasis (Ahrends et al., 2022). To assess the diversity of the predictions, we examined the correlation between predictions generated by distinct HMMs to indicate if they potentially contained differential information. Additionally, we examined the accuracy of these predictions to discern whether diversity was driven by inaccurate predictions, or whether they were distinct and complementary. By combining diverse yet accurate predictions (e.g., multiple predictions which are accurate, but each one explains one aspect of the data better than the others), we aimed to develop accurate subject-specific trait predictions. This approach also offers the advantage of circumventing the challenge of model selection by combining the information from multiple representations of the data.

### 2.7 Performance evaluation

We evaluated predictions for 16,352 subjects from UKB, and 1,001 subjects from HCP, or as many subjects as data were available for a given trait. To investigate the effectiveness of stacking, we compared the performance of the stacked prediction with the base-level predictions that were combined to generate it. We also compared our stacked prediction with the common and most simplistic form of combination, which involves taking the average of all base-level predictions so that each one contributes equally to the final prediction (Lincoln & Skrzypekt, 1989; Perrone & Cooper, 1992; Tumer & Ghosht, 1996a). In other words, given k base-level predictions, ${\hat{y}}_{k}$, we combine them as follows:

$$\text{Simple averaging:}\;{\hat{y}}_{average}=\;\;\frac{1}{N}\sum_{k=1}^{N}\;{\hat{y}}_{k}.$$ (13)

Our models were assessed based on accuracy and robustness. Accuracy was measured using the coefficient of determination, R^2^ (see Supplementary Methods 1.3 for details).

For a model to be robust, the model’s accuracy should be consistent across variations of the training set. Robustness is extremely important for real-world applications, but often a shortcoming in neuroimaging-based prediction studies (Varoquaux et al., 2017).

We estimated prediction accuracy using 10-fold cross-validation. For each trait, we repeated the cross-validation process 10 times using different randomised folds, following the approach of Džeroski & Ženko (2004). This allowed us to assess the consistency and variance of our models’ accuracy. To assess accuracy, we calculated the mean R^2^ scores across the 10 cross-validation iterations. To assess robustness, we examined the variance of the R^2^ scores across the 10 iterations.

To assess the statistical significance of the differences in accuracy between our different approaches, we used the corrected repeated k-fold cross-validation test (Bouckaert & Frank, 2004), where we used r=10 repeats of k=10 fold cross-validation.

For a given fold i and repetition j of the cross-validation, let the accuracy of two prediction approaches be $a_{ij}$ and $b_{ij}$ , and their difference be $x_{ij}\;=a_{ij}\;-b_{ij}$ . The corrected repeated k-fold *t*-statistic is given by

$$t=\;\frac{\frac{1}{k\cdot r}\sum_{i=1}^{k}\;\sum_{j=1}^{r}x_{ij}}{\sqrt{\frac{1}{k\cdot r\;}+\;\frac{n_{2}}{n_{1}}{\hat{\sigma }}^{2}}},$$ (14)

where $n_{1}$ and $n_{2}$ are the number of training and test instances, respectively, and ${\hat{\sigma }}^{2}$ is an estimate for the variance of the differences given by

$${\hat{\sigma }}^{2}=\frac{1}{k\cdot r-1}\sum_{i=1}^{k}\overset{r}{\underset{j=1}{\;\sum }}\left(x_{ij}-\frac{1}{k\cdot r}\sum_{i=1}^{k}\overset{r}{\underset{j=1}{\;\sum }}x_{ij}\right).$$ (15)

We compared the mean prediction accuracies across 15 subject traits for each dataset separately. To account for multiple comparisons (i.e., the 30 different tests composed of the 15 comparisons between different stacking approaches for the 2 datasets), we applied Benjamini-Hochberg’s False Discovery Rate (FDR) procedure (Benjamini & Hochberg, 1995) to correct the resulting p-values.

To investigate the robustness of stacking, we compared the variance of the distribution of accuracies across cross-validation iterations between base-level predictions and stacked predictions. Since the distributions did not follow a normal distribution for certain traits (see Supplementary Table SI-4 for details), we used Levene’s test to compare the distributions. Similar to our previous analysis, p-values were corrected across using Benjamini-Hochberg’s FDR procedure (across the 60 different tests comprising 15 from UKB and 15 from HCP tests for the 2 sets of Levene’s tests).

## 3 Results

In this section, we investigate the effectiveness of stacking predictions using HMMs with fixed hyperparameters and HMMs with varying hyperparameters. We find that stacking predictions obtained from HMMs generally improve prediction accuracy, although its effectiveness compared with alternative prediction approaches depends on the dataset. We then showcase the flexibility and power of stacking by combining predictions from static FC with those from dynamic FC. Furthermore, we show that stacking can improve the robustness of predictions, but that the success of stacking compared with simpler model averaging depends on sample size. Finally, we explore the factors contributing to the effectiveness of stacking in certain scenarios.

The results presented in the subsequent section focus on predictions generated using the methodological steps outlined above. Specifically, we model data from rfMRI timeseries obtained from an ICA50 parcellation using an HMM that focuses on both the FC between regions and their activation patterns. We then estimate precision matrices from the covariance matrices representing the functional connectivity between brain regions, and project these onto the tangent space before generating predictions. Each of these steps represents a methodological choice that influences the subsequent predictions. In Supplementary Figure SI-3, we provide additional analyses exploring alternative options. These options include using covariances (as opposed to partial correlations) and using the Fisher kernel method.

### 3.1 Stacking predictions generated from HMMs with varying hyperparameters can improve prediction accuracy

Our analysis revealed that combining predictions generated from 50 HMMs with fixed hyperparameters (i.e., those with run-to-run variability caused by different initialisations and the stochasticity of the HMM inference) resulted in a higher average prediction accuracy than base-level predictions for UKB but was generally ineffective for HCP.

**Fig. 3.**
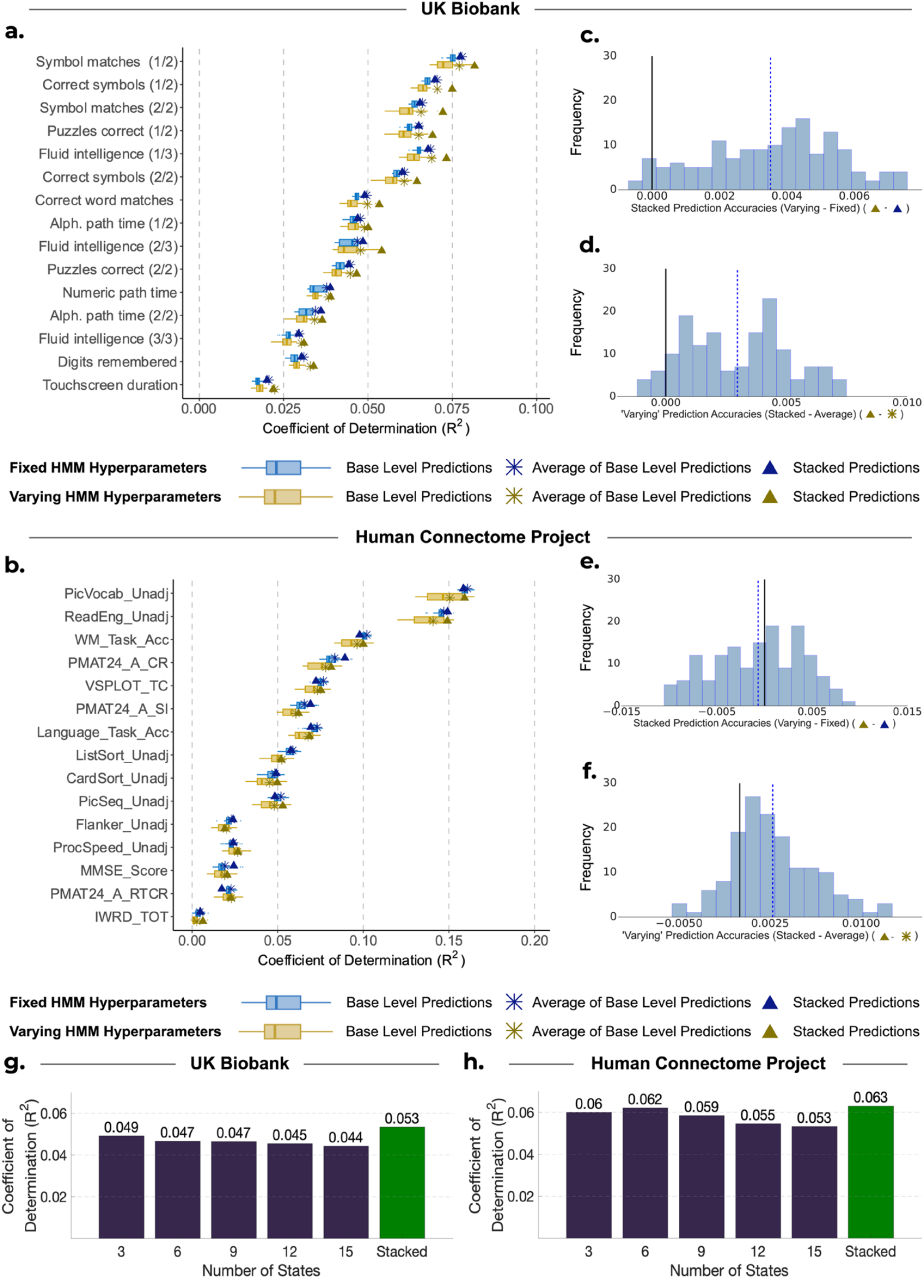
Comparison of performance for base-level predictions and stacking predictions from HMMs with varying hyperparameters against HMMs with fixed hyperparameters. (a, b) Performance of stacking across subject traits for UKB and HCP, respectively. Boxplots show the R^2^ scores between observed subject traits and base-level predictions generated from 50 HMMs. These are compared with the R^2^ scores when we combine the base-level predictions by taking the average of them (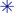) and by stacking (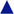). Blue represents the results of using HMMs with fixed hyperparameters. Yellow represents the results of using HMMs with varying model hyperparameters. (c, e) Distribution of the difference between stacking (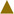) and averaging (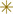) predictions from HMMs with varying hyperparameters across 10 cross-validation iterations and 15 cognitive traits for (c) UKB and (e) HCP. (d, f) Distribution of the difference between stacking prediction using varying (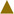) and fixed (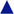) hyperparameters across 10 cross-validation iterations and 15 cognitive traits for (d) UKB and (f) HCP. (g, h) Each bar represents the mean accuracy of base-level predictions across 10 cross-validation repetitions and base-level predictions obtained from HMMs with the stated number of states.

Figure 3 provides a comparison of the distribution of explained variance (coefficients of determination; R^2^) between predictions generated from HMMs with fixed hyperparameters (depicted in blue) and observed subject traits for three approaches: base-level predictions (shown by the boxplots), averaging base-level predictions (shown by the crosses), and stacking them using constrained least squares (shown by the triangles). The respective comparison between predictions generated from HMMs with varying hyperparameters (depicted in yellow) and observed traits is also shown.

The results for the UKB are depicted in Figure 3a. Across 15 traits and 10 cross-validation repetitions, stacking predictions from HMMs with fixed hyperparameters exhibited a higher average prediction accuracy compared with base-level predictions (showing a statistically significant but very modest improvement), but performed equally to simply averaging across base-level predictions (mean R^2^ STACKED: 0.0524 vs. mean R^2^ BASE: 0.0497, *p_BH_* < 0.01; vs. mean R^2^ AVERAGE: 0.0525, *p_BH_* = 0.374). On the other hand, stacking predictions obtained from HMMs with varying hyperparameters exhibited small improvements over both the base-level predictions and simple averaging (mean R^2^ STACKED: 0.0559 vs. mean R^2^ BASE: 0.0488, *p_BH_* < 0.01; vs. mean R^2^ AVERAGE: 0.0529; *p_BH_* < 0.01; see Fig. 3c). The stacked prediction consistently outperformed every base-level prediction for all subject traits, highlighting the benefit of stacking over selecting a single configuration of the HMM for predictions for UKB. Furthermore, stacking predictions from HMMs with varying hyperparameters was slightly more accurate than stacking predictions from HMMs with fixed hyperparameters (mean R^2^ VARY: 0.0559 vs. mean R^2^ FIXED: 0.0524, *p_BH_* < 0.01). Figure 3d shows the difference in these R^2^ scores, highlighting this small but consistent improvement across traits.

Figure 3b shows the accuracy values for HCP. Across all subject traits, stacking predictions from HMMs with fixed hyperparameters performed similarly to base-level predictions and averaging across base-level predictions (mean R^2^ STACKED: 0.0456 vs. mean R^2^ BASE: 0.0444, *p_BH_* = 0.144; vs. mean R^2^ AVERAGE: 0.0460, *p_BH_* = 0.663). While stacking predictions from HMMs with varying hyperparameters was significantly better than base-level predictions (mean R^2^ STACKED: 0.0443 vs. mean R^2^ BASE: 0.0396, *p_BH_* < 0.01), stacking did not significantly outperform simply averaging across base-level predictions (mean R^2^ STACKED: 0.0443 vs. mean R^2^ AVERAGE: 0.0424; *p_BH_* = 0.122; see Fig. 3e).

Furthermore, stacking predictions from HMMs with varying hyperparameters did not produce more accurate predictions than stacking predictions from HMMs with fixed hyperparameters (mean R^2^ VARY: 0.0443 vs. mean R^2^ FIXED: 0.0456; *p_BH_* = 0.248; see Fig. 3f). To explore this further, we found the mean prediction accuracy across all cross-validation repetitions and base-level predictions corresponding to each number of HMM states (for the varying hyperparameter case). The findings are depicted in Figure 3g for UKB and Figure 3h for HCP, showing that predictions from HMMs with six states were the most accurate for HCP, while predictions from HMMs with three states yielded the highest accuracy for UKB. Therefore, if we had chosen an alternative configuration for developing HMMs with fixed hyperparameters, our results would likely have been different. Given that a different number of states were optimal for HCP and UKB, it is, therefore, recommended to use stacking to determine the best HMM configuration, or combination of configurations, rather than trying to select a configuration a priori.

To assess the impact of de-confounding, we replicated the analysis without adjusting for confounding effects. Supplementary Figure SI-4 presents the results of this analysis.

### 3.2 Dynamic predictions outperform static predictions with sufficient data

While dynamic models, such as the HMMs above, can potentially capture more information, predictions based on static FC may show an advantage due to their simplicity and reliability. Our goal for this analysis was to investigate if predictions from static FC can complement, or improve upon, predictions from dynamic FC. Given that the case of varying hyperparameter (exhibiting both run-to-run variability and hyperparameter selection variability) subsumes the case of fixed hyperparameters (run-to-run variability only), we, therefore, focus solely on predictions from dynamic FC generated from HMMs with varying hyperparameters.

**Fig. 4.**
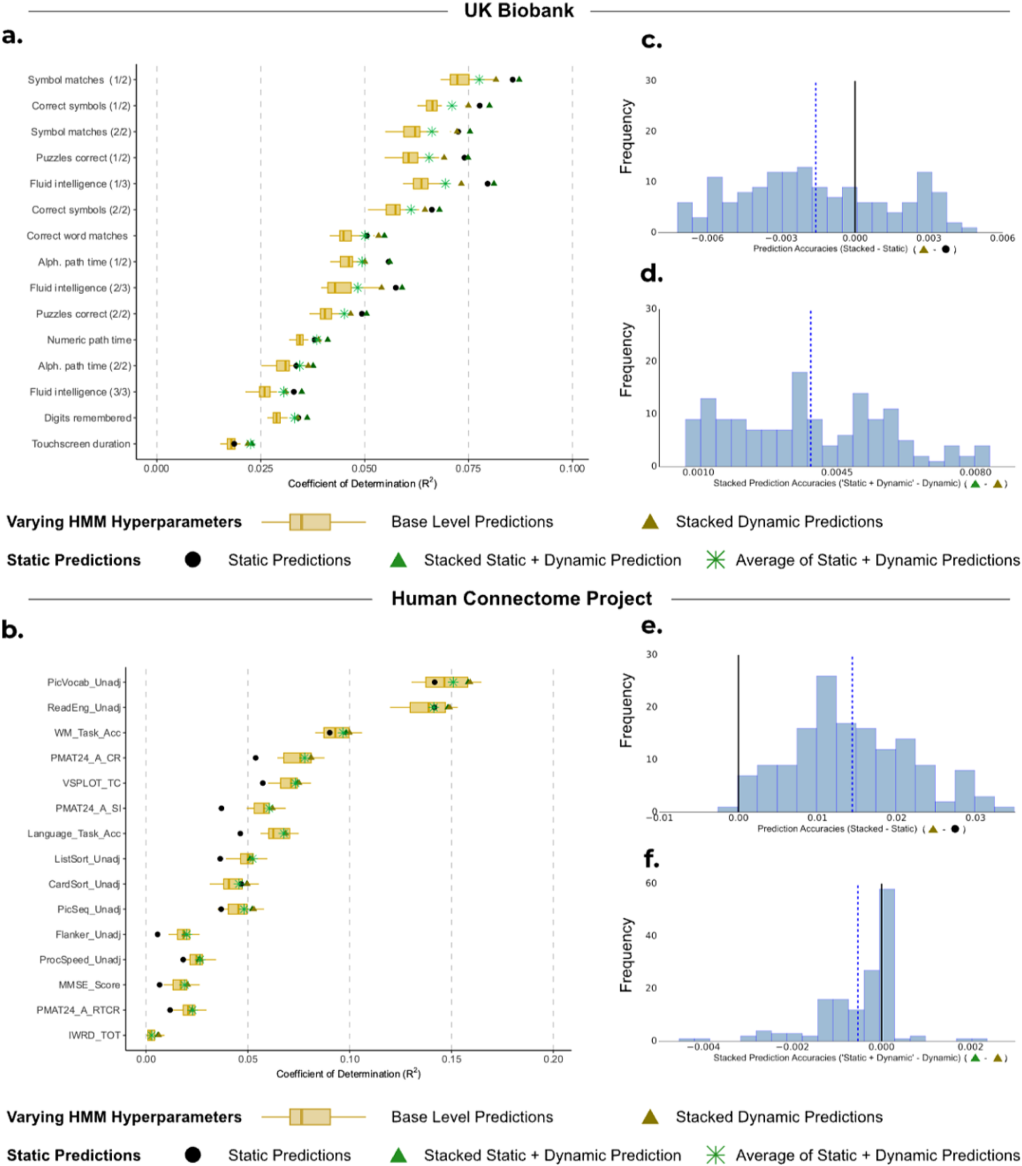
Comparison of performance for static FC, base-level dynamic FC predictions, and stacking the predictions. (a, b) Boxplots show the R^2^ scores between observed subject traits and dynamic base-level predictions generated from 50 HMMs and are compared with predictions from static FC (•). These individual predictions are then compared with the R^2^ scores when we combine the base-level predictions by stacking the dynamic base-level predictions with varying HMM hyperparameters (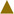), as well as stacking the predictions from static FC with dynamic base-level predictions (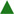) for (a) UKB and (b) HCP. (c, e) Distribution of the difference between stacking prediction using varying hyperparameters (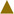) and predictions from static FC (•) across 10 cross-validation iterations and 15 cognitive traits for (c) UKB and (e) HCP. (d, f) Distribution of the difference between stacking predictions from static FC with predictions from dynamic FC using varying hyperparameters (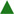) and stacking predictions from dynamic FC only (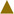) across 10 cross-validation iterations and 15 cognitive traits for (d) UKB and (f) HCP.

For UKB, Figure 4a shows that predictions from static FC outperformed the stacked predictions from dynamic FC (mean R^2^ STATIC: 0.0575 vs. mean R^2^ DYNAMIC (STACKED): 0.0559; *p_BH_* = 0.0463; see Fig. 4c). However, the combination of static and dynamic base-level predictions using our stacking framework (i.e., the 50 dynamic base-level predictions and 1 prediction from static FC) resulted in a significant improvement in accuracy compared with static predictions alone (mean R^2^ STATIC+DYNAMIC (STACKED): 0.0598 vs. mean R^2^ STATIC: 0.0574, *p_BH_* < 0.01), as well as compared with stacking only the predictions from dynamic FC (mean R^2^ STATIC+DYNAMIC (STACKED): 0.0598 vs. mean R^2^ DYNAMIC (STACKED): 0.0559, *p_BH_* < 0.01; see Fig. 4d).

Moreover, this combined static and dynamic approach also outperformed the simpler method of averaging across the 51 predictions (mean R^2^ STATIC+DYNAMIC (STACKED): 0.0598 vs. mean R^2^ STATIC+DYNAMIC (AVERAGE): 0.0533; *p_BH_* < 0.01). These results highlight a benefit of stacking compared with simple model averaging: the ability to distinguish a single prediction that significantly outperforms all the other predictions (here, the single prediction from static FC), and potentially improve upon it.

For HCP, Figure 4b highlights that the stacked prediction from dynamic FC significantly outperformed predictions from static FC (mean R^2^ VARYING (STACKED): 0.0443 vs. mean R^2^ STATIC: 0.0301, *p_BH_* < 0.01; Fig. 4e). Stacking the predictions from dynamic FC together with those from static FC resulted in comparable accuracies to only stacking the predictions from dynamic FC (see Fig. 4f), as well as averaging across dynamic and static predictions (mean R^2^ DYNAMIC (STACKED): 0.0444 vs. mean R^2^ STATIC+DYNAMIC (STACKED): 0.0443, *p_BH_* = 0.975; vs. mean R^2^ STATIC+DYNAMIC (AVERAGE): 0.0426, *p_BH_* = 0.193).

Overall, our findings suggest that dynamic FC tends to generate more accurate predictions than static FC when there is sufficient high-quality data and longer scans. The HCP dataset, with its 4,800 time points compared with UKB’s 490, provided ample data for dynamic FC to yield superior prediction accuracy. Conversely, in UKB, where scanning sessions were shorter, the predictions from static FC outperformed those from dynamic FC for the traits we examined.

To explore any potential difference in effects of confounds for static FC and dynamic FC, we also replicated this analysis without accounting for confounding effects. The result of this analysis is shown in Supplementary Figure SI-5.

### 3.3 Stacking leads to a more robust prediction with sufficient sample size

**Fig. 5.**
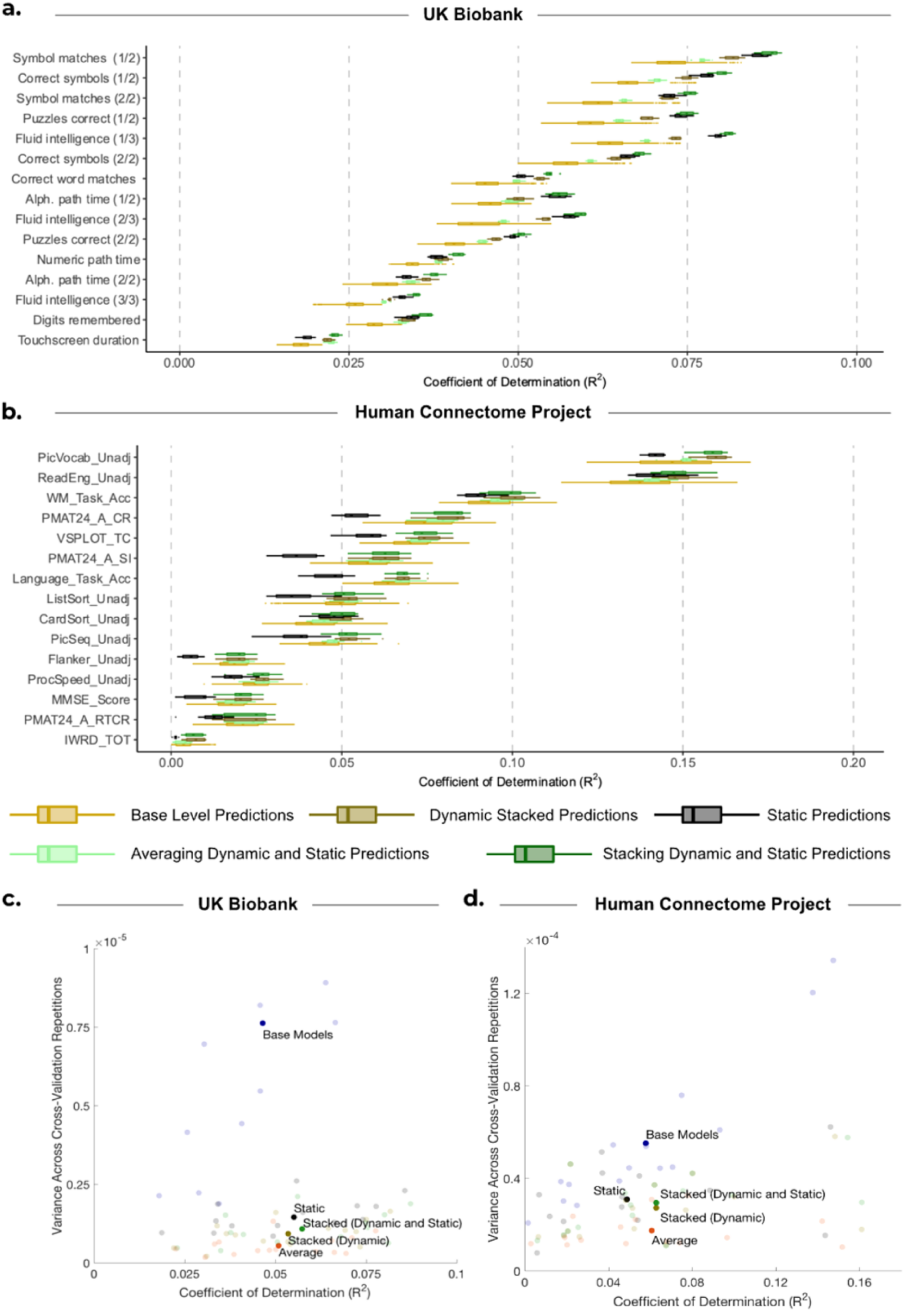
Comparison of accuracy and robustness for different prediction approaches. (a) UK Biobank and (b) Human Connectome Project. “Base-level Predictions” boxplots (light yellow) show the R^2^ scores between observed subject traits and 500 base-level predictions (50 HMMs across 10 cross-validation iterations). These are compared with the predictions across 10 cross-validation iterations for predictions from static FC (black), stacked predictions from dynamic FC only (dark gold), averaging across predictions from dynamic and static FC (light green), and stacked predictions from dynamic FC and static FC together (dark green). (c, d) Comparison of accuracy and robustness across prediction approaches for (c) UK Biobank. (d) Human Connectome Project. For each approach, accuracy is measured as the mean R^2^ score across 10 cross-validation repetitions, and robustness is measured as the variance across 10 cross-validation repetitions. The dark bold circles represent the mean across all 15 subject traits, and the transparent circles represent the underlying 15 subject traits which average out to give the dark bold circle.

While diversity among base-level predictions can result in improved stacked predictions (e.g., through varying the HMM hyperparameters), it is important to ensure that diversity is not driven by a lack of robustness, where model accuracies vary depending on the subjects used for training. To assess the robustness of the predictions when subjects were randomised across cross-validation folds, we conducted 10 cross-validation iterations independently for 50 HMMs with varying hyperparameters. Figure 5a and b compares robustness across prediction approaches, and Figure 5c and d presents a summary of the performance of the methods in terms of both robustness and accuracy.

For UKB, Figure 5a compares the variance in accuracy values for the various prediction approaches. For each approach, the figure presents prediction accuracies across cross-validation iterations (i.e., full repeats of the nested cross-validation framework with different randomised fold distributions). Compared with the base-level predictions, stacking predictions from HMMs with varying hyperparameters (yellow and gold boxplots) significantly improved robustness across cross-validation iterations for 11 of the 15 traits (p_BH_ < 0.05^**^). We focused our statistical analysis on the comparison of robustness between base-level predictions and stacking predictions from dynamic FC since this is the focus of this study; however, Figure 5c suggests that model averaging, as well as predictions from static FC, was also much more robust than base-level predictions. The figure highlights that, for UKB, although the alternative approaches also improve robustness, stacking prediction from both dynamic FC and static FC exhibited the best accuracy while also being robust.

For HCP, stacking was ineffective at producing robust predictions, as shown in Figure 5b. Stacking predictions obtained from HMMs with varying hyperparameters (yellow and gold boxplots) only increased robustness for 2 out of 15 subject traits (*p_BH_* < 0.05^**^). Figure 5d highlights that for HCP, stacking and averaging exhibit a similar level of accuracy but averaging provides a more robust prediction. The disparity between the two datasets can be attributed to the larger number of subjects in UKB, resulting in stacked predictions outperforming averaging in UKB. Conversely, the smaller number of subjects in HCP resulted in higher variability across cross-validation iterations for stacking.

### 3.4 Stacking and model averaging are comparable with limited subjects

**Fig. 6.**
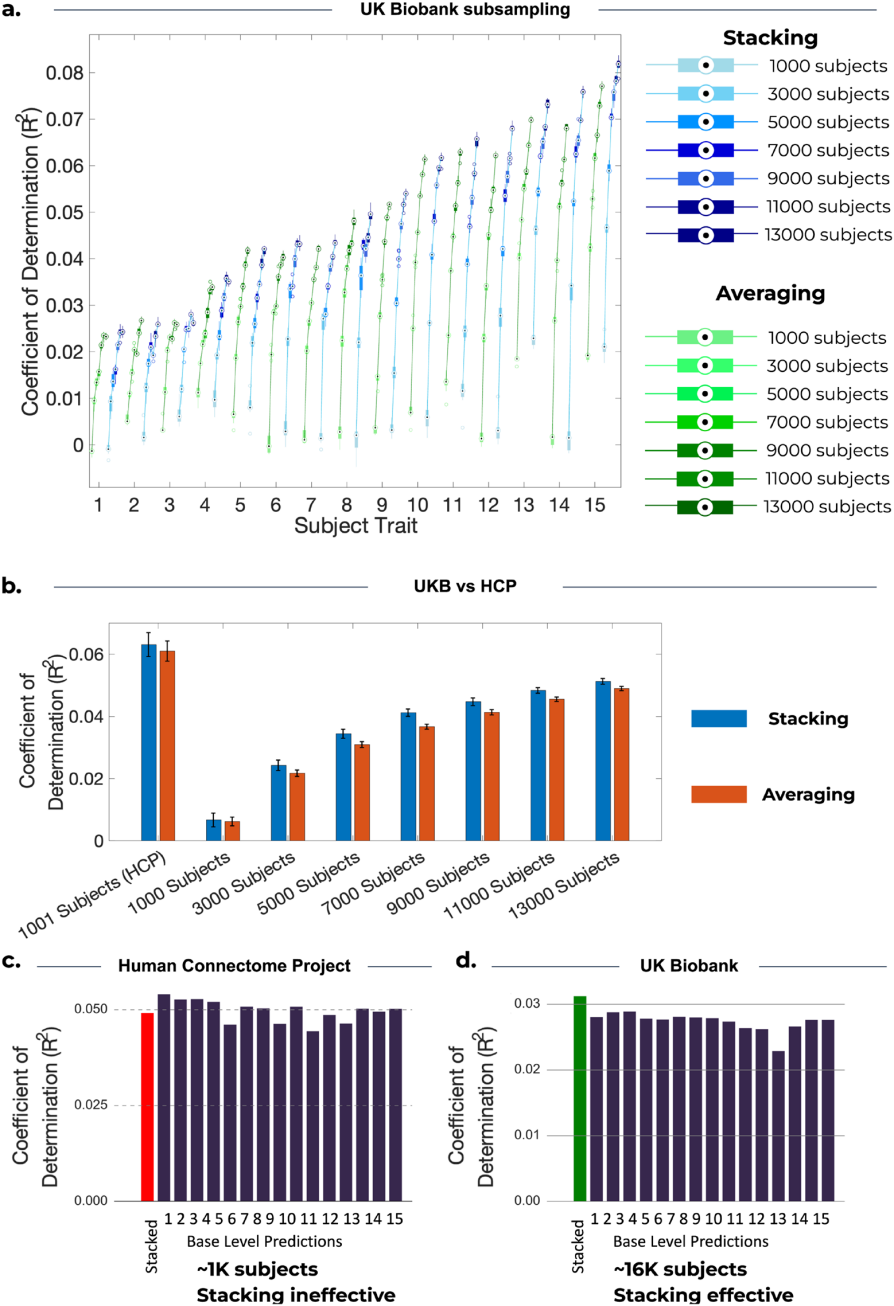
Comparison of stacking and model averaging across 10 cross-validation iterations for different sample sizes. (a) Each boxplot represents the R^2^ scores across 10 cross-validation iterations for the corresponding subject trait and for the given sample size. As sample size increases, prediction accuracies increase, and stacking outperforms model averaging. (b) Each bar represents the mean R^2^ scores across 10 cross-validation iterations and 15 subject traits. Error bars are calculated by finding the standard deviation across the cross-validation iterations for each subject trait, and then taking the mean across these 15 traits. The left pair of bars represents the accuracy for 1,001 subjects from the HCP dataset, and the remaining bars show the results for an increasing number of subjects from UKB. (c, d) The bottom panel of the figure shows the accuracy of base-level predictions (R^2^) for the 15 base-level predictions corresponding to the largest stacking weights from the pool of 50 models for predicting example subject traits in HCP and UKB. (c) HCP: NIH Toolbox Dimensional Change Card Sort Test: Unadjusted Scale Score (“CardSort_Unadj”). (d) UKB: Number of fluid intelligence questions attempted within time limit (2.0) (“Fluid intelligence (3/3)”). In contrast to UKB, the limited number of subjects in HCP prevents stacking from producing a superior stacked prediction.

To explore the relationship between the effectiveness of stacking and sample size, we generated stacked predictions for UKB across a range of sample sizes. The result of our analysis is shown in Figure 6, where we present prediction accuracies for sample sizes from 1,000 subjects, to match HCP, up to 15,000 subjects^††^.

Figure 6a shows that the accuracy of stacked predictions continued to increase with larger sample sizes, up to 15,000 subjects. Furthermore, Figure 6b highlights that we are unable to conclude that stacking outperforms model averaging for 1,000 subjects, but as sample size increases, there is more evidence that stacking outperforms averaging. This suggests that it is due to the smaller sample size in HCP that stacking performs similarly to averaging. Figure 6c shows, for one chosen trait in HCP, that stacking has a prediction accuracy that is inferior to many base-level predictions. Conversely, for UKB, Figure 6d shows that stacking can produce a prediction which outperforms all base-level predictions. This disparity suggests that the estimation process of the stacking weights can be hindered by statistical noise. Specifically, the stacking process may assign non-zero stacking weights to less accurate predictions, which is undesirable. This can be caused by certain models performing well during training on specific subjects but performing poorly on the test set, leading to a stacked prediction less accurate than some base-level predictions. With a sufficient sample size (such as in the case of UKB), stacking is expected to perform at least as well as the best base-level predictions. This is because the stacking process could, at the very least, assign a stacking weight of 1 to the best base-level predictions, effectively disregarding the remaining predictions, whereas model averaging cannot.

### 3.5 Stacking is effective when base-level predictions are diverse yet accurate

But, when is stacking most useful? To effectively stack multiple base-level predictions, the predictions should both be diverse and show a minimum level of accuracy (Hansen & Salamon, 1990). One way to signify diversity is by seeking predictions that are less correlated with each other, indicating differential information (Tumer & Ghosht, 1996b). We found that varying hyperparameters of the HMM generally led to more diversity and subsequently a better stacked prediction. We have seen that predictions in HCP lack robustness, where variability can be driven by statistical noise, therefore, we focus on UKB for this analysis.

**Fig. 7.**
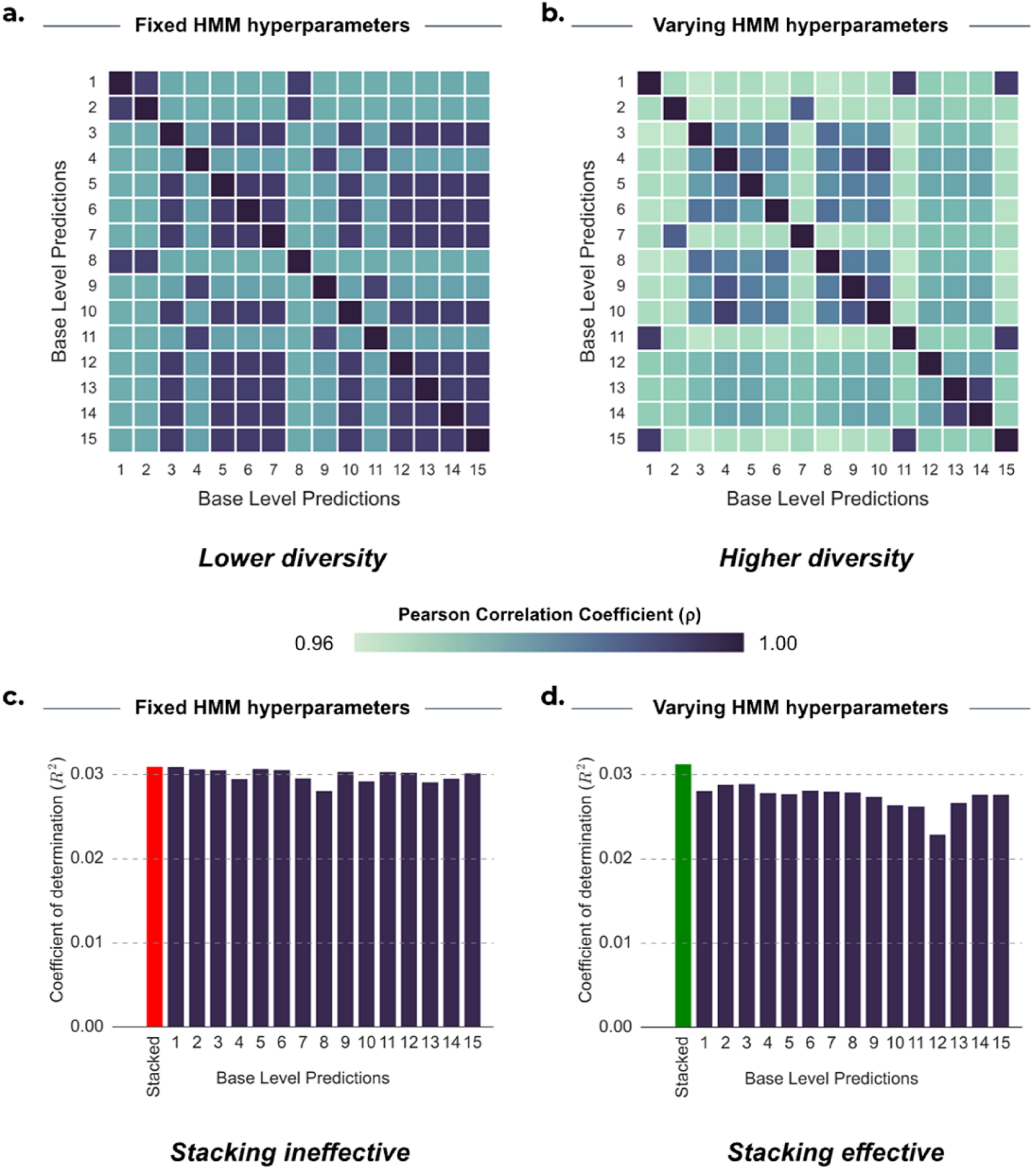
Diversity and accuracy analyses of 15 base-level predictions corresponding to the largest stacking weights from the pool of 50 models for predicting 2 cognitive traits from UKB. In the top panel, correlation between base-level predictions is shown for (a) Maximum digits remembered correctly (2.0) (Digits remembered) and (b) Number of fluid intelligence questions attempted within time limit (2.0) (Fluid intelligence (3/3)). In the bottom panel, the accuracy of base-level predictions (R^2^) is shown for (c) Digits remembered and (d) Fluid intelligence (3/3). The predictions were all generated from HMMs with varying hyperparameters. Higher diversity in the base-level predictions resulted in more effective stacking for fluid intelligence.

We explored the differences in diversity induced by both run-to-run variability and hyperparameter selection variability. As an illustrative example, Figure 7 provides a summary of our findings on the impact of both types of variability of the HMM for two representative cognitive traits in UKB; “Maximum digits remembered correctly” (Digits remembered) and “Number of fluid intelligence questions attempted within time limit” (Fluid intelligence (3/3)). We observed that base-level predictions from HMMs with fixed hyperparameters (Fig. 7a; mean ρ_FIXED_: 0.958) exhibited higher correlations with each other compared with predictions from HMMs with varying hyperparameters (Fig. 7b; mean ρ_VARY_: 0.931).

Greater diversity (indicated by lower correlation values) generally led to improved stacked predictions. Figure 7c shows that when the hyperparameters of the HMM were fixed, producing highly correlated predictions, there was not enough variability for stacking to produce a more accurate prediction than many of the base-level predictions (mean R^2^ STACK: 0.0310; mean R^2^ BASE: 0.0300; best R^2^ BASE: 0.0310). However, Figure 7d shows that when the hyperparameters of the HMM are varied, stacking yielded a higher accuracy than all base-level predictions (mean R^2^ STACK: 0.0345; mean R^2^ BASE: 0.0292; best R^2^ BASE: 0.0306). Despite the fact that many of the best-performing base-level predictions displayed similar levels of accuracy for many traits in UKB, it is the diversity (as indicated by lower correlation between predictions) that drives successful stacking. Nonetheless, it is important to ensure that this diversity stems from complementary and meaningful patterns in the data rather than inaccurate predictions (i.e., pure noise), as seems to be the case in HCP.

Furthermore, compared with averaging, stacking is useful when a subset of base-level predictions performs notably better than the rest. For instance, predictions from static FC tended to outperform those from dynamic FC in UKB, resulting in a stacked prediction that yielded superior predictions to straightforward model averaging. Additionally, stacking is effective in scenarios where certain initialisations of the HMM produce significantly more accurate base-level predictions than others.

In Figure 3a, the more accurate base-level predictions are represented as the outliers for several subject traits^‡‡^, represented by small dots to the right-hand side of the respective boxplots. Stacking is particularly effective for these traits since stacking can disregard the less accurate predictions, where simple model averaging cannot. In these scenarios, stacking is providing a data-driven way of selecting the best HMM hyperparameters or determining whether predictions from static FC or dynamic FC perform best.

## 4 Discussion

Generating robust and accurate predictions of subject traits (e.g., clinical or psychological) from brain data is crucial for both the interpretation and the clinical translation of findings. Previous research has shown that dynamic (time-varying) representations of the brain can add information above and beyond that of static (time-averaged) functional representations (Liégeois et al., 2019; Vidaurre et al., 2021), resulting in superior prediction accuracies for behavioural traits using brain dynamics (Ahrends et al., 2024). To complement structural representations of the brain, and potentially improve over static FC representations, we employed generative models of brain network dynamics and extracted features suitable for subject trait prediction. By combining predictions from multiple brain dynamic models that provided distinct and complementary perspectives of the data, we presented two benefits of stacking. Firstly, we showed that in certain scenarios, stacked predictions were slightly more accurate and robust across cross-validation iterations than base-level predictions; this is consistent with previous work on model combination for statistical testing (Vidaurre et al., 2019). Secondly, we highlighted how this approach eliminates the need to arbitrarily select the “best” HMM hyperparameters, and instead incorporates information from many options, frequently generating superior predictions for certain subject traits.

We found that stacking base-level predictions obtained from HMMs with fixed hyperparameters (i.e., those with run-to-run variability) failed to improve prediction accuracy compared with the base-level predictions and simple model averaging for both HCP and UKB. This outcome is not surprising, as the variation among HMMs with identical hyperparameters but different initialisations is likely to be limited and driven by the difference in the quality of estimations rather than differences in information content. However, we also found that by exploring a range of HMM hyperparameters, we generated diverse base-level predictions that were combined to produce superior stacked predictions for UKB. Previous research has shown that the selection of HMM hyperparameters can influence the sensitivity to different temporal scales in FC changes (Ahrends et al., 2022), and this information can be optimally combined for prediction using our approach. We found that base-level predictions obtained from HMMs with varying hyperparameters were found to be less correlated with each other, indicating diversity, and were, therefore, more likely to produce more accurate stacked prediction.

Overall, these findings indicate that by varying the hyperparameters of the HMM, we go beyond merely reducing noise through combining predictions towards an effective combination of complementary views of the data. However, it is essential to acknowledge that the usefulness of stacking may be limited in studies with small-to-moderate sample sizes—at least for traits that exhibit a moderate prediction accuracy such as the ones contemplated here. Indeed, the potential of stacking is inherently dependent on the phenotypes under study. As our ability to predict phenotypes, such as cognitive traits, improves, stacking is likely to become increasingly beneficial.

The objective of our research was to evaluate the effectiveness of stacking compared with alternative approaches. In doing so, our approach used all subjects to develop the ICA-based brain parcellation for HCP, and all subjects for HMM training for both datasets. In other words, unsupervised screening steps were performed before test subjects were left out. Critically, this does not give the predictors an unfair advantage, first because all the considered approaches are compared on the same grounds, and second because, as discussed in Hastie et al. (2008), we do not use the target features of the test set. Regarding the second point, if the primary objective of our research was to generalise to unseen samples without re-training the model on the entire dataset, then it is important to restrict all pre-processing, including HMM training, to each training fold within the cross-validation framework. This would also be important if we would not have access to the original data at the time of prediction. Furthermore, we have previously explored the differences in accuracy resulting from performing HMM training within cross-validation folds compared with across training and test subjects in the HCP dataset, and the results were comparable (Vidaurre et al., 2021). This is consistent with previous literature indicating that the effects of data leakage (from such unsupervised preprocessing steps) on predictive performance in neuroimaging datasets are negligible for larger sample sizes (Rosenblatt et al., 2023) (as opposed, for example, to the dangers from feature selection and re-using subjects in the training and test set).

It is important to appreciate the differences between the two considered datasets. In UKB, there are a large number of subjects, but a relatively short scanning time per subject, whereas in HCP we have fewer subjects, but they underwent longer scanning sessions. This resulted in two differences. Firstly, our predictions in UKB were more robust, whereas the accuracy of predictions in HCP was more susceptible to changes in training examples. This finding is consistent with previous research, which has shown that large sample sizes are necessary for replicable brain–phenotype associations, particularly for intelligence traits (Liu et al., 2023). Therefore, while it is still worth generating and subsequently combining multiple base-level predictions, simple model averaging is recommended in datasets with a sample size similar to HCP. Secondly, the longer scanning session in HCP resulted in dynamic FC adding valuable information beyond that of static FC, whereas prediction from static FC outperformed those from dynamic FC in UKB. This suggests that to reveal unique information beyond that of static FC when using existing dynamic FC techniques, a dataset with sufficient quantity and quality of data per subject, as found in HCP, is more appropriate. Despite this, the combination of predictions from both static and dynamic FC was most accurate in UKB, illustrating the strength of stacking different features for predictions.

In this study, our predictions from dynamic FC were developed by projecting precision matrices onto the tangent space. Previous research has demonstrated the superior performance of the Fisher kernel compared with naïve kernel approaches that directly use HMM features (Ahrends et al., 2024). However, our use of full covariances between brain regions with the Fisher kernel resulted in less accurate predictions than other approaches that were based on precision matrices (see Supplementary Fig. SI-3). We anticipate that incorporating precision matrices into the Fisher kernel approach will improve prediction accuracy compared with alternative approaches, although this will be explored in future research. Additionally, potential future avenues include further diversification of base-level predictions and stacking methods. For example, by applying the HMM to different timeseries from the same group of subjects, analyses from multiple brain imaging modalities (e.g., M/EEG) and/or different brain parcellations could be combined using nonlinear stacking approaches (e.g. random forests).

Overall, our proposed method offers a consistent and flexible approach to integrating multiple models of brain dynamics, demonstrating that combining complementary models of brain dynamics allows us to potentially achieve more accurate and robust predictions. The stacking framework we introduced not only provides flexibility for future exploration, but also reduces the risk of poor predictions due to trivial factors such as poor initialisations, incorrect hyperparameter choice, or cross-validation structure.

## Supplementary Material

Supplementary Material

## Data Availability

The data used in this manuscript pertain to two well-known, open-access repositories: the Human Connectome Project dataset and the UK Biobank dataset. The code used in this study was implemented in MATLAB 2019a. The MATLAB code for the analysis is publicly available in the repository of the HMM-MAR toolbox at https://github.com/OHBA-analysis/HMM-MAR, and Python equivalents are publicly available at https://github.com/vidaurre/glhmm and https://github.com/OHBA-analysis/osl-dynamics.

## Footnotes

* The initial state probabilities, π, are excluded from the prediction analysis since they contain minimal informative information for subject trait predictions at the expense of extra computation.

† In the HMM-MAR MATLAB toolbox and the glhmm Python toolbox, this is specified by the “DirichletDiag” and “dirichlet_diag” options, respectively.

‡ K=6,δ=10.

§ K∈{3, 6, 9, 12, 15}, δ∈{10, 100, 1000, 10000, 100000}. See Supplementary Table SI-3 for full details.

** See Supplementary Table SI-5 for p-values for each trait.

††

N= 1000, 3000, 5000, 7000, 9000, 11000, 13000, 15000.

‡‡ See, for example, Number of puzzles correctly solved (Puzzles correct (1/2)), Number of symbol digit matches made correctly (Correct symbols (1/2)), Number of symbol digit matches made attempted (Symbol matches (2/2)), and Fluid intelligence score (Fluid intelligence (1/3)).
