# Supplementary material for: Stacking models of brain dynamics to improve prediction of subject traits in fMRI
Source: Imaging Neurosci (Camb). 2024 Aug 20;2:imag-2-00267. doi: 10.1162/imag_a_00267 (PMC11886951; doi:10.1162/imag_a_00267)
Supplement: Supplementary Material [file imag_a_00267-supp.pdf]

# Ben Griffin, Christine Ahrends, Chetan Gohil, Fidel Alfaro-Almagro, Mark W. Woolrich, Stephen M. Smith, Diego Vidaurre (2024): Stacking models of brain dynamics to improve prediction of subject traits in fMRI

## Supplementary Methods

### 1.1 The Fisher kernel

In addition to the other methods described for predicting subject traits, we explored the Fisher kernel method (Ahrends et al., 2024; Jaakkola et al., 2000; Jaakkola & Haussler, 1998), which is a mathematically principled approach to predict from generative probabilistic models. The method proceeds by calculating the Fisher scores, $U_x$, given by the gradient of the log-likelihood with respect to each parameter:

$$U_{x_t} = \nabla \mathcal{L}_\theta(x_t),$$

where $x_t$ is the fMRI timeseries of a given subject, and $\mathcal{L}_\theta(x_t) = P(x_t|\theta)$ is the log-likelihood of timeseries $x_t$ given the HMM parameters $\theta$.

The invariant Fisher kernel, $K_I$, is then defined as:

$$K_I(x_m, x_n) = U_{x_m}^T \mathcal{F}^{-1} U_{x_n}$$

for subjects $m$ and $n$, where $\mathcal{F}$ is the Fisher information matrix defined by:

$$\mathcal{F} = \mathbb{E}_x[U_x^T \cdot U_x],$$

which accounts for the different scales of the various model parameters.

In practice, the Fisher information matrix is often disregarded because its impact is negligible as sample size increases while being computationally expensive (Jaakkola & Haussler, 1998; Shawe-Taylor & Cristianini, 2004).

As a result, the practical Fisher kernel, $K_F$, is often used, for which the linear version is defined as:

$$K_F(x_m, x_n) = U_{x_m}^T \cdot U_{x_n}.$$

The dot product is computed for all subject pairs to calculate a kernel matrix, which can be used with any suitable kernel prediction method. In our study, we adopted a linear kernel in combination with kernel ridge regression.

## 1.2 Optimising state-specific partial correlation calculations

The optimal level of regularisation required to generate state-specific partial correlation matrices depends on the number of states of the HMM. Given an HMM with $K$ states, we determine the optimal $a$ by taking each state $k$ in turn, and first noting down the covariance matrix $\Sigma_{\mathrm{k}}$, for all subjects. We then calculate the root mean square distance between the regularised precision matrix for each subject, and the group average of the unregularized precision matrix across all subjects for a grid of regularisation values[1]. We take the mean of this distance across all subjects for all possible levels of regularisation, from which a singular $a$ is selected. After repeating this for each state, we take the mean of the optimal values across all states, to determine a single $a$ for a given HMM of $K$ states. This value is used for all subjects.

This was repeated for all HMMs with different numbers of states (i.e., 3, 6, 9, 12, 15), since we anticipated that HMMs with more states may require different levels of regularization since, on average, less data is used for determining the covariance matrices and as such, the estimates may be noisier.

## 1.3 Coefficient of Determination

Given an observed subject trait, $y$, and our prediction, $\hat{y}$, for $N$ subjects, the coefficient of determination is given by:

$$R^2 = 1 - \frac{\sum_{i=1}^{N}(y_i - \hat{y}_i)^2}{\sum_{i=1}^{N}(y_i - \bar{y})^2}$$

where $\bar{y}$ is the mean of the observed subject trait over all subjects.

[1] $a = (0, 0.01, 0.02, \ldots, 1)$.

## Supplementary Material

***Table SI-1*** *HCP Subject Traits.*

| Var. no. | Column Header | Full Display Name | Assessment | HCP Var. no. |
|---|---|---|---|---|
| 1 | ReadEng_Unadj | NIH Toolbox Oral Reading Recognition Test: Unadjusted Scale Score | Language/Reading | 231 |
| 2 | PicVocab_Unadj | NIH Toolbox Picture Vocabulary Test: Unadjusted Scale Score | Language/Vocabulary | 233 |
| 3 | WM_Task_Acc | Working Memory Task OVERALL Accuracy | Working Memory Task | 545 |
| 4 | PMAT24_A_CR | Penn Progressive Matrices: Number of Correct Responses (PMAT24_A_CR) | Fluid Intelligence | 228 |
| 5 | ListSort_Unadj | NIH Toolbox List Sorting Working Memory Test: Unadjusted Scale Score | Working Memory | 264 |
| 6 | PMAT24_A_SI | Penn Progressive Matrices: Total Skipped Items (PMAT24_A_SI) | Fluid Intelligence | 229 |
| 7 | PicSeq_Unadj | NIH Toolbox Picture Sequence Memory Test: Unadjusted Scale Score | Episodic Memory | 222 |
| 8 | VSPLOT_TC | Variable Short Penn Line Orientation: Total Number Correct (VSPLOT_TC) | Spatial Orientation | 251 |
| 9 | CardSort_Unadj | NIH Toolbox Dimensional Change Card Sort Test: Unadjusted Scale Score | Executive Function/ Cognitive Flexibility | 224 |
| 10 | Language_Task_Acc | Language Task OVERALL Accuracy | Language Task | 510 |
| 11 | Flanker_Unadj | NIH Toolbox Flanker Inhibitory Control and Attention Test: Unadjusted Scale Score | Executive Function/ Inhibition | 226 |
| 12 | IRWD_TOT | Penn Word Memory Test: Total Number of Correct Responses (IWRD_TOT) | Verbal Episodic Memory | 262 |
| 13 | ProcSpeed_Unadj | NIH Toolbox Pattern Comparison Processing Speed Test: Unadjusted Scale Score | Processing Speed | 235 |
| 14 | PMAT_24_A_RTCR | Penn Progressive Matrices: Median Reaction Time for Correct Responses (PMAT24_A_RTCR) | Fluid Intelligence | 230 |
| 15 | MMSE_SCORE | Mini Mental Status Exam Total Score | Cognitive Status (Mini Mental Status Exam) | 196 |

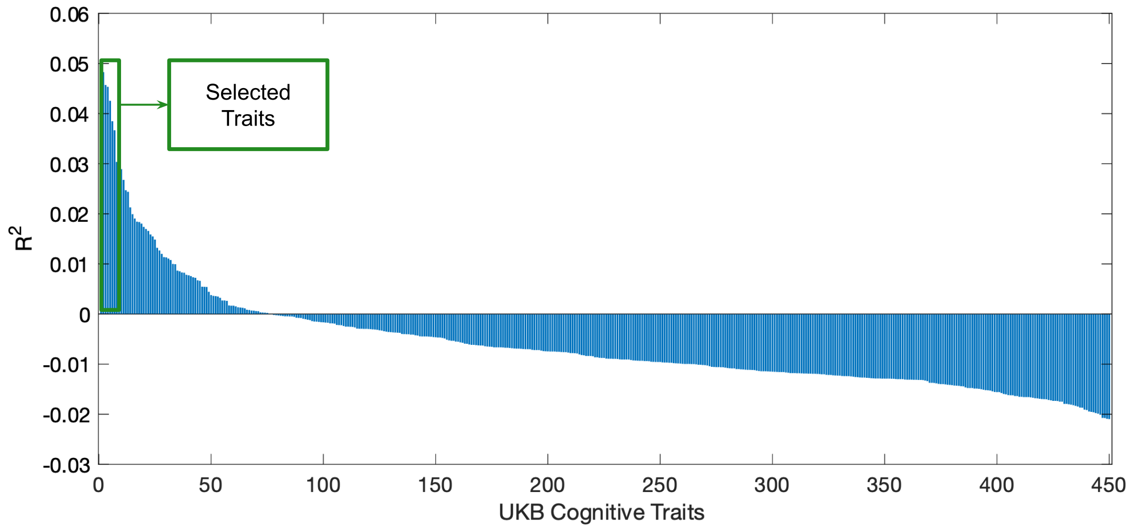


***Figure SI-1*** *Coefficient of determination ($R^2$) between observed subject traits and predictions generated from static FC for the 450 cognitive traits in UKB for which we performed preliminary analysis to select 15 subject traits for further investigation.*

***Table SI-2*** *UKB Subject Traits.*

| Var. no. | Column Header | Full Display Name | Category | UKB Field ID |
|---|---|---|---|---|
| 1 | Symbol matches (1/2) | Number of symbol digit matches attempted (2.0) | Symbol digit substitution | 23323 |
| 2 | Puzzles correct (1/2) | Number of puzzles correctly solved (2.0) | Matrix pattern completion | 6373 |
| 3 | Correct symbols (1/2) | Number of symbol digit matches made correctly (2.0) | Symbol digit substitution | 23324 |
| 4 | Fluid intelligence (1/3) | Fluid intelligence score (2.0) | Fluid intelligence / reasoning (cognitive function summary) | 20016 |
| 5 | Symbol matches (2/2) | Number of symbol digit matches made attempted (0.0) | Symbol digit substitution | 23323 |
| 6 | Correct symbols (2/2) | Number of symbol digit matches made correctly (0.0) | Symbol digit substitution | 23324 |
| 7 | Alph. path time (1/2) | Duration to complete alphanumeric path (trail #2) (0.0) | Trail making | 6350 |
| 8 | Correct word matches | Number of word pairs correctly associated (2.0) | Paired associate learning | 20197 |
| 9 | Fluid intelligence (2/3) | Fluid intelligence score (0.0) | Fluid intelligence / reasoning (cognitive function summary) | 20016 |
| 10 | Puzzles correct (2/2) | Number of puzzles correct (2.0) | Tower rearranging | 21004 |
| 11 | Numeric path time | Duration to complete numeric path (trail #1) (2.0) | Trail making | 6348 |
| 12 | Alph. path time (2/2) | Duration to complete alphanumeric path (trail #2) (2.0) | Trail making | 6350 |
| 13 | Fluid intelligence (3/3) | Number of fluid intelligence questions attempted within time limit (2.0) | Fluid intelligence / reasoning (cognitive function summary) | 20128 |
| 14 | Digits remembered | Maximum digits remembered correctly (2.0) | Numeric memory | 4282 |
| 15 | Touchscreen duration | Touchscreen duration (2.0) | Process duration | 21622 |

Notes
(0.0) refers to the initial assessment visit (2006-2010) at which participants were recruited and consent given.
(2.0) refers to the first imaging visit (2014+)

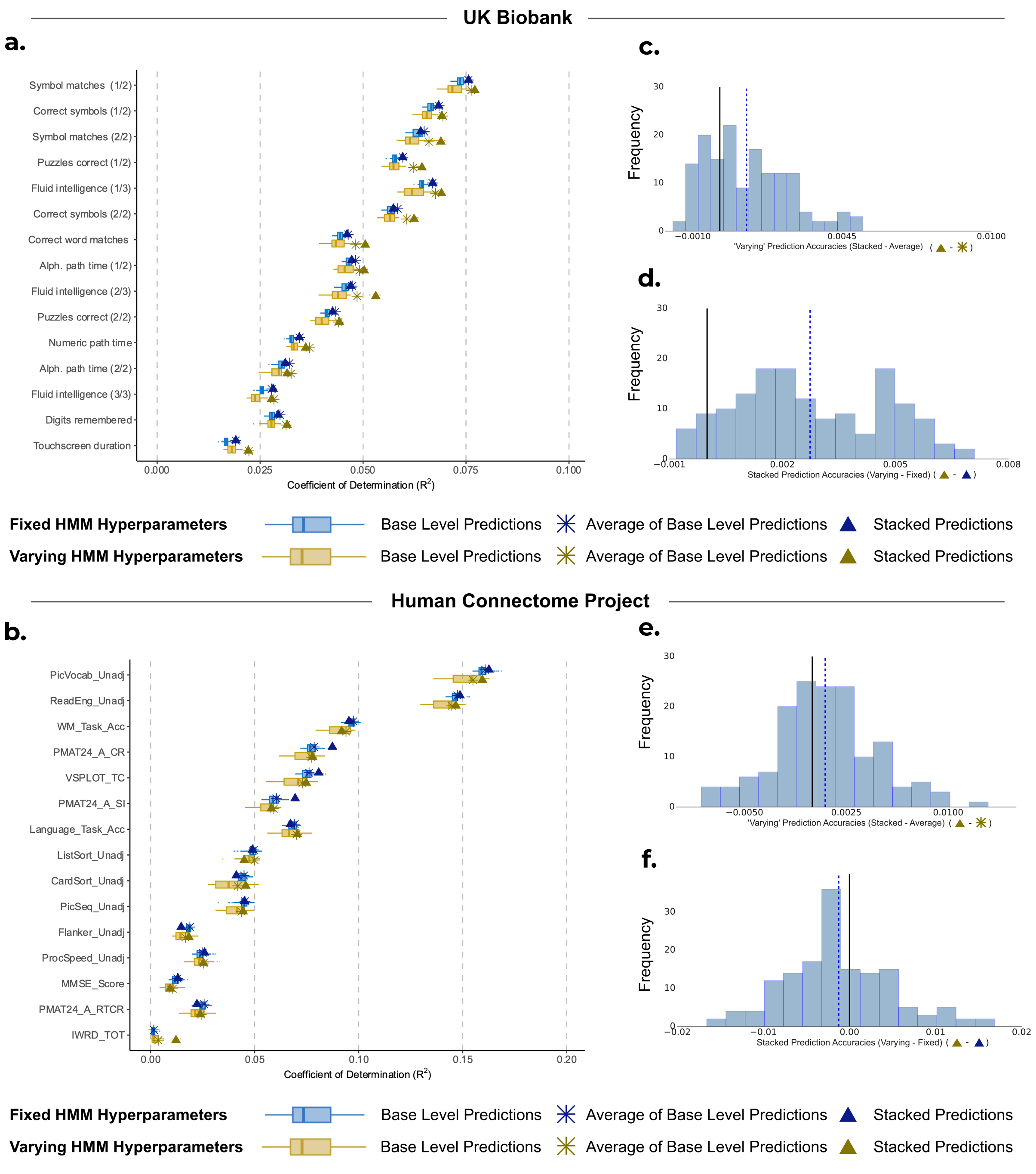


***Figure SI-2** Comparison of performance for base-level predictions and stacking predictions from HMMs with varying hyperparameters against HMMs with fixed hyperparameters, where all HMMs model FC only.* **(a), (b)** Performance of stacking across subject traits for UKB and HCP respectively. Boxplots show the $R^2$ scores between observed subject traits and base-level predictions generated from 50 HMMs. These are compared to the $R^2$ scores when we combine the base-level predictions by taking the average of them (✳) and by stacking (▲). Blue represents the results of using HMMs with fixed hyperparameters. Yellow represents the results of using HMMs with varying model hyperparameters. **(c)** Distribution of the difference between stacking predictions (▲) and averaging predictions (✳) using varying hyperparameters across 10 cross-validation iterations and 15 cognitive traits for UKB (left) and HCP (right).

***Table SI-3*** *Choice of HMM hyperparameters for the 50 HMMs where they were varied.*

| HMM run | Number of states ($K$) | Dirichlet distribution concentration parameter ($\delta$) |
|---|---|---|
| 1 | 3 | 10 |
| 2 | 3 | 100 |
| 3 | 3 | 1000 |
| 4 | 3 | 10000 |
| 5 | 3 | 100000 |
| 6 | 6 | 10 |
| 7 | 6 | 100 |
| 8 | 6 | 1000 |
| 9 | 6 | 10000 |
| 10 | 6 | 100000 |
| 11 | 9 | 10 |
| 12 | 9 | 100 |
| 13 | 9 | 1000 |
| 14 | 9 | 10000 |
| 15 | 9 | 100000 |
| 16 | 12 | 10 |
| 17 | 12 | 100 |
| 18 | 12 | 1000 |
| 19 | 12 | 10000 |
| 20 | 12 | 100000 |
| 21 | 15 | 10 |
| 22 | 15 | 100 |
| 23 | 15 | 1000 |
| 24 | 15 | 10000 |
| 25 | 15 | 100000 |
| 26 | 3 | 10 |
| 27 | 3 | 100 |
| 28 | 3 | 1000 |
| 29 | 3 | 10000 |
| 30 | 3 | 100000 |
| 31 | 6 | 10 |
| 32 | 6 | 100 |
| 33 | 6 | 1000 |
| 34 | 6 | 10000 |
| 35 | 6 | 100000 |
| 36 | 9 | 10 |
| 37 | 9 | 100 |
| 38 | 9 | 1000 |
| 39 | 9 | 10000 |
| 40 | 9 | 100000 |
| 41 | 12 | 10 |
| 42 | 12 | 100 |
| 43 | 12 | 1000 |
| 44 | 12 | 10000 |
| 45 | 12 | 100000 |
| 46 | 15 | 10 |
| 47 | 15 | 100 |
| 48 | 15 | 1000 |
| 49 | 15 | 10000 |
| 50 | 15 | 100000 |

***Table SI-4*** *p-values for one-sample Kolmogorov-Smirnov test assessing if the (standardised) distribution of accuracy values ($R^2$; coefficients of determination) for the base-level and stacked predictions for traits in UKB and HCP come from a normal distribution.*

| **Dataset** | **HMM Hyperparameter Type** | **Var. no.** | **Column Header** | **p-vals** Base-Level Prediction | **p-vals** Stacked Prediction |
|---|---|---|---|---|---|
| UKB | Fixed | 1 | Symbol matches (1/2) | 0.4448 | 0.776 |
| UKB | Fixed | 2 | Puzzles correct (1/2) | 0.6784 | 0.8721 |
| UKB | Fixed | 3 | Correct symbols (1/2) | 0.1531 | 0.5489 |
| UKB | Fixed | 4 | Fluid intelligence (1/3) | 0.5895 | 0.9679 |
| UKB | Fixed | 5 | Symbol matches (2/2) | 0.7753 | 0.7004 |
| UKB | Fixed | 6 | Correct symbols (2/2) | 0.7988 | 0.9605 |
| UKB | Fixed | 7 | Alph. path time (1/2) | 0.7191 | 0.6336 |
| UKB | Fixed | 8 | Correct word matches | 0.4915 | 0.9869 |
| UKB | Fixed | 9 | Fluid intelligence (2/3) | 0.0648 | 0.6612 |
| UKB | Fixed | 10 | Puzzles correct (2/2) | 0.838 | 0.9979 |
| UKB | Fixed | 11 | Numeric path time | <0.0001* | 0.8408 |
| UKB | Fixed | 12 | Alph. path time (2/2) | 0.0097 | 0.5217 |
| UKB | Fixed | 13 | Fluid intelligence (3/3) | 0.8622 | 0.8217 |
| UKB | Fixed | 14 | Digits remembered | 0.8163 | 0.9993 |
| UKB | Fixed | 15 | Touchscreen duration | 0.0618 | 0.7377 |
| UKB | Vary | 1 | Symbol matches (1/2) | 0.0554 | 0.95 |
| UKB | Vary | 2 | Puzzles correct (1/2) | 0.0076 | 0.7784 |
| UKB | Vary | 3 | Correct symbols (1/2) | 0.1641 | 0.541 |
| UKB | Vary | 4 | Fluid intelligence (1/3) | 0.0011* | 0.8222 |
| UKB | Vary | 5 | Symbol matches (2/2) | 0.001* | 0.9341 |
| UKB | Vary | 6 | Correct symbols (2/2) | 0.006* | 0.762 |
| UKB | Vary | 7 | Alph. path time (1/2) | 0.0532 | 0.8707 |
| UKB | Vary | 8 | Correct word matches | 0.259 | 0.9826 |
| UKB | Vary | 9 | Fluid intelligence (2/3) | 0.006* | 0.8914 |
| UKB | Vary | 10 | Puzzles correct (2/2) | 0.0011* | 0.9814 |
| UKB | Vary | 11 | Numeric path time | 0.0017* | 0.9627 |
| UKB | Vary | 12 | Alph. path time (2/2) | 0.3609 | 0.982 |
| UKB | Vary | 13 | Fluid intelligence (3/3) | 0.0007* | 0.9579 |
| UKB | Vary | 14 | Digits remembered | 0.5598 | 0.7284 |
| UKB | Vary | 15 | Touchscreen duration | 0.396 | 0.9674 |
| HCP | Fixed | 1 | ReadEng_Unadj | <0.0001* | 0.8496 |
| HCP | Fixed | 2 | PicVocab_Unadj | 0.0042* | 0.8183 |
| HCP | Fixed | 3 | WM_Task_Acc | 0.0062* | 0.7994 |
| HCP | Fixed | 4 | PMAT24_A_CR | 0.0014* | 0.9668 |
| HCP | Fixed | 5 | ListSort_Unadj | <0.0001* | 0.9645 |
| HCP | Fixed | 6 | PMAT24_A_SI | <0.0001* | 0.6162 |
| HCP | Fixed | 7 | PicSeq_Unadj | <0.0001* | 0.5139 |
| HCP | Fixed | 8 | VSPLOT_TC | 0.009* | 0.9471 |
| HCP | Fixed | 9 | CardSort_Unadj | 0.0012* | 0.7162 |
| HCP | Fixed | 10 | Language_Task_Acc | <0.0001* | 0.7765 |
| HCP | Fixed | 11 | Flanker_Unadj | 0.1245 | 0.9184 |
| HCP | Fixed | 12 | IRWD_TOT | 0.0043* | 0.8959 |
| HCP | Fixed | 13 | ProcSpeed_Unadj | <0.0001* | 0.6316 |
| HCP | Fixed | 14 | PMAT_24_A_RTCR | 0.003* | 0.7453 |
| HCP | Fixed | 15 | MMSE_SCORE | 0.005* | 0.3752 |
| HCP | Vary | 1 | ReadEng_Unadj | 0.0212* | 0.6563 |
| HCP | Vary | 2 | PicVocab_Unadj | 0.015* | 0.9213 |
| HCP | Vary | 3 | WM_Task_Acc | <0.0001* | 0.9608 |
| HCP | Vary | 4 | PMAT24_A_CR | 0.0787 | 0.6251 |
| HCP | Vary | 5 | ListSort_Unadj | <0.0001* | 0.8127 |
| HCP | Vary | 6 | PMAT24_A_SI | <0.0001* | 0.5418 |
| HCP | Vary | 7 | PicSeq_Unadj | <0.0001* | 0.6412 |
| HCP | Vary | 8 | VSPLOT_TC | <0.0001* | 0.8011 |
| HCP | Vary | 9 | CardSort_Unadj | <0.0001* | 0.9712 |
| HCP | Vary | 10 | Language_Task_Acc | 0.3198 | 0.2797 |
| HCP | Vary | 11 | Flanker_Unadj | <0.0001* | 0.923 |
| HCP | Vary | 12 | IRWD_TOT | 0.0178* | 0.9667 |
| HCP | Vary | 13 | ProcSpeed_Unadj | <0.0001* | 0.9838 |
| HCP | Vary | 14 | PMAT_24_A_RTCR | <0.0001* | 0.851 |
| HCP | Vary | 15 | MMSE_SCORE | 0.145 | 0.2992 |

*statistically significant results

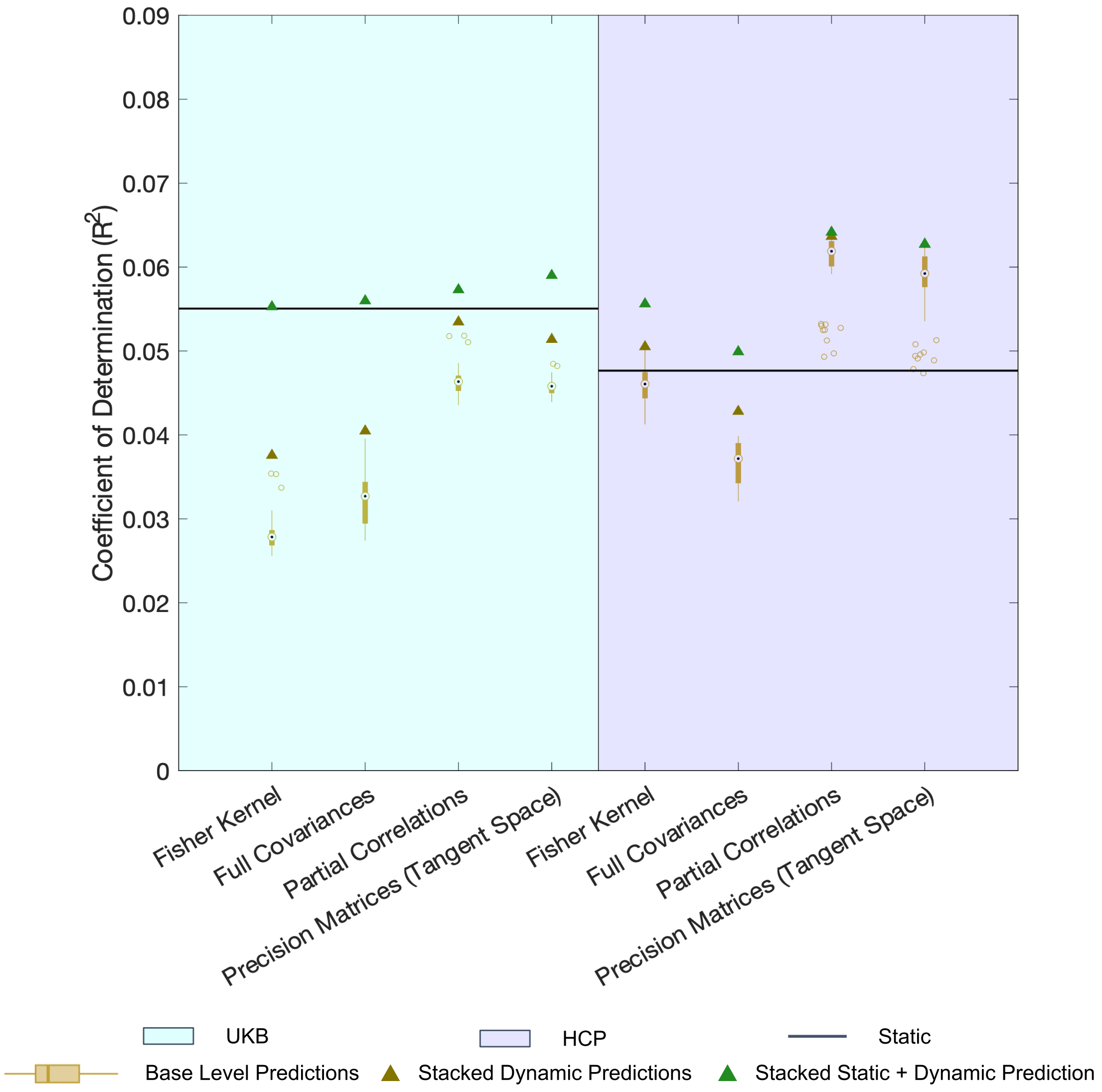


***Figure SI-3*** *Comparison of performance across different prediction approaches (predictions from static FC are compared to predictions from dynamic FC using the Fisher kernel method, and with those developed from full covariances, partial correlations, and projecting precision matrices onto tangent space). UKB is displayed on the left-hand side (light blue) and HCP is displayed on the right-hand side (light purple).* For each prediction approach, boxplots show the $R^2$ scores between observed subject traits and dynamic base-level predictions generated from 50 HMMs and are compared to predictions from static FC (black line). These individual predictions are then compared to the $R^2$ scores when we combine the base-level predictions by stacking the dynamic base level predictions with varying HMM hyperparameters (▲), as well as stacking the static predictions with dynamic base level predictions (▲).

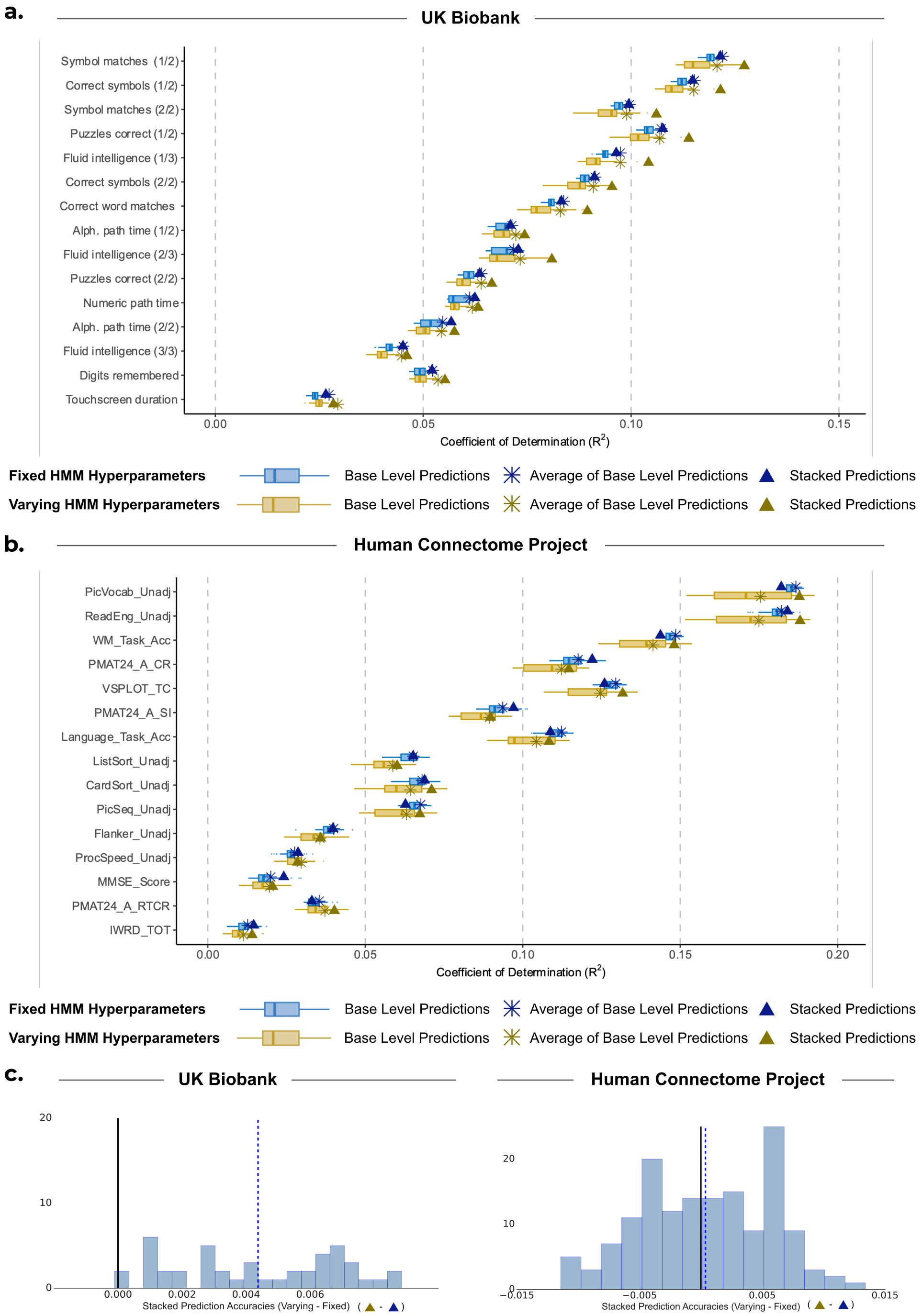


***Figure SI-4** Comparison of performance for base-level predictions and stacking predictions from HMMs with varying hyperparameters against HMMs with fixed hyperparameters without deconfounding.* **(a), (b)** Performance of stacking across subject traits for UKB and HCP respectively. Boxplots show the $R^2$ scores between observed subject traits and base-level predictions generated from 50 HMMs. These are compared to the $R^2$ scores when we combine the base-level predictions by taking the average of them (✳) and by stacking (▲). Blue represents the results of using HMMs with fixed hyperparameters. Yellow represents the results of using HMMs with varying model hyperparameters. **(c)** Distribution of the difference between stacking predictions (▲) and averaging predictions (✳) using varying hyperparameters across 10 cross-validation iterations and 15 cognitive traits for UKB (left) and HCP (right).

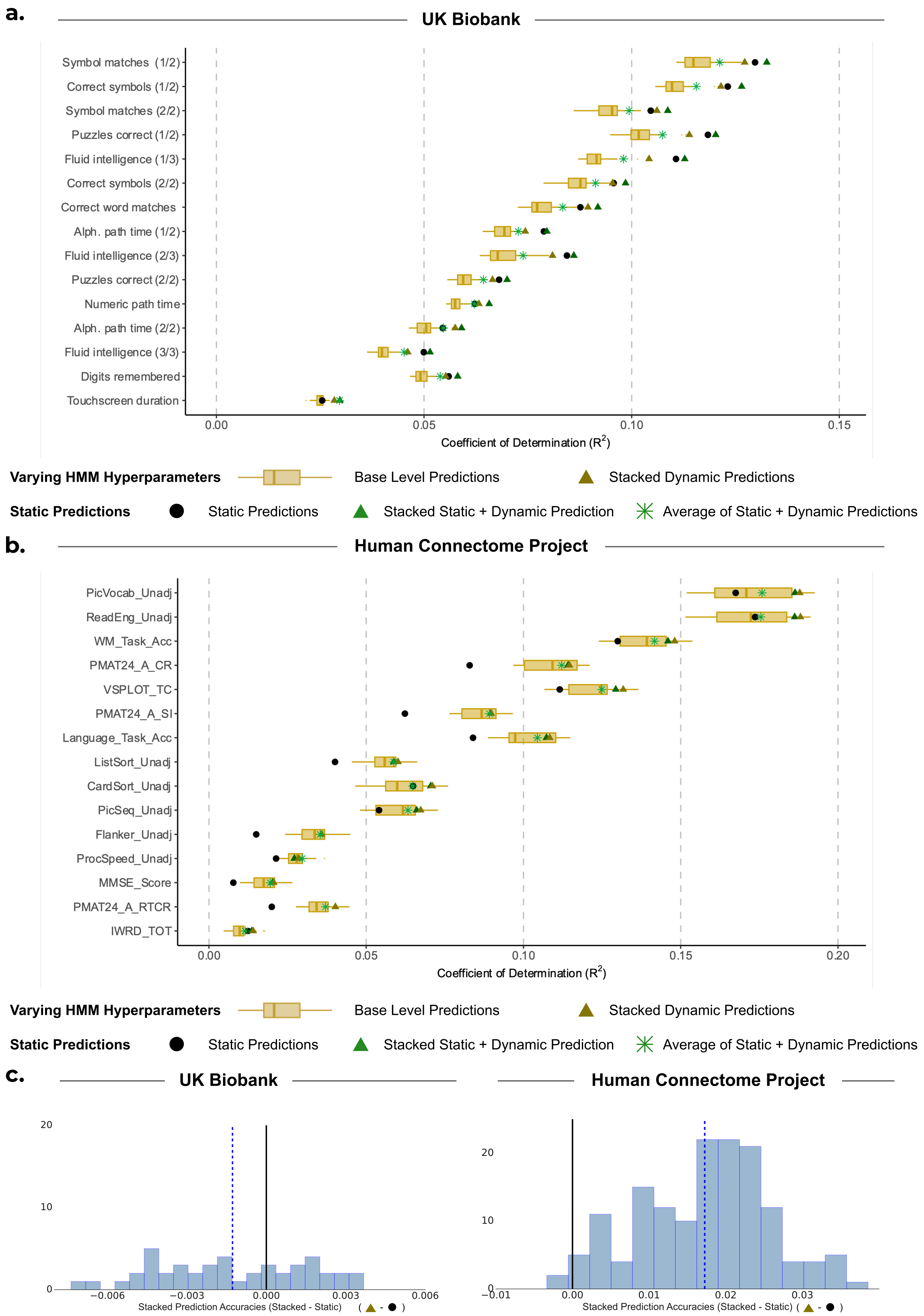


***Figure SI-5** Comparison of performance for static FC, base-level dynamic FC predictions, and stacking the predictions without deconfounding.* **(a)** UKB. **(b)** HCP. Boxplots show the $R^2$ scores between observed subject traits and dynamic base-level predictions generated from 50 HMMs and are compared to static FC predictions (●). These individual predictions are then compared to the $R^2$ scores when we combine the base-level predictions by stacking the dynamic base level predictions with varying HMM hyperparameters (▲), as well as stacking the static predictions with dynamic base level predictions (▲). While predictions from static FC using partial correlation network matrices outperform the predictions from dynamic FC using full correlation matrices for both datasets, combining the static and dynamic predictions together results in the most accurate predictions in UKB.

***Table SI-5*** *p-values for Levene's tests comparing the differences in variance between base level predictions and stacked prediction for UKB and HCP subject traits. To account for multipe comparisons, we applied Benjamini-Hochberg's False Discovery Rate procedure to correct the p-values within each datset separately.*

| **Dataset** | **HMM Hyperparameter Type** | **Var. no.** | **Column Header** | **Variance** Base-Level Prediction (x e-05) | **Variance** Stacked Prediction (x e-05) | **p-value** |
|---|---|---|---|---|---|---|
| UKB | Fixed | 1 | Symbol matches (1/2) | 0.1381 | 0.0196 | 0.043* |
| UKB | Fixed | 2 | Puzzles correct (1/2) | 0.2284 | 0.1184 | 0.294 |
| UKB | Fixed | 3 | Correct symbols (1/2) | 0.3199 | 0.2285 | 0.549 |
| UKB | Fixed | 4 | Fluid intelligence (1/3) | 0.2036 | 0.1378 | 0.399 |
| UKB | Fixed | 5 | Symbol matches (2/2) | 0.2206 | 0.1134 | 0.333 |
| UKB | Fixed | 6 | Correct symbols (2/2) | 0.1827 | 0.0662 | 0.231 |
| UKB | Fixed | 7 | Alph. path time (1/2) | 0.2663 | 0.1977 | 0.615 |
| UKB | Fixed | 8 | Correct word matches | 0.4214 | 0.1564 | 0.299 |
| UKB | Fixed | 9 | Fluid intelligence (2/3) | 0.3668 | 0.1681 | 0.231 |
| UKB | Fixed | 10 | Puzzles correct (2/2) | 0.4115 | 0.2370 | 0.275 |
| UKB | Fixed | 11 | Numeric path time | 0.3579 | 0.1772 | 0.346 |
| UKB | Fixed | 12 | Alph. path time (2/2) | 0.6276 | 0.2453 | 0.231 |
| UKB | Fixed | 13 | Fluid intelligence (3/3) | 0.1943 | 0.1210 | 0.459 |
| UKB | Fixed | 14 | Digits remembered | 0.3085 | 0.2032 | 0.615 |
| UKB | Fixed | 15 | Touchscreen duration | 0.2217 | 0.1113 | 0.275 |
| UKB | Vary | 1 | Symbol matches (1/2) | 0.0242 | 0.0020 | 0.030* |
| UKB | Vary | 2 | Puzzles correct (1/2) | 0.0272 | 0.0133 | 0.440 |
| UKB | Vary | 3 | Correct symbols (1/2) | 0.1184 | 0.0161 | 0.013* |
| UKB | Vary | 4 | Fluid intelligence (1/3) | 0.0336 | 0.0121 | 0.160 |
| UKB | Vary | 5 | Symbol matches (2/2) | 0.2086 | 0.0108 | 0.009* |
| UKB | Vary | 6 | Correct symbols (2/2) | 0.1800 | 0.0101 | 0.007* |
| UKB | Vary | 7 | Alph. path time (1/2) | 0.0593 | 0.0128 | 0.089 |
| UKB | Vary | 8 | Correct word matches | 0.0889 | 0.0076 | 0.011* |
| UKB | Vary | 9 | Fluid intelligence (2/3) | 0.0822 | 0.0165 | 0.039* |
| UKB | Vary | 10 | Puzzles correct (2/2) | 0.1793 | 0.0158 | 0.007* |
| UKB | Vary | 11 | Numeric path time | 0.1026 | 0.0208 | 0.059 |
| UKB | Vary | 12 | Alph. path time (2/2) | 0.0707 | 0.0161 | 0.039* |
| UKB | Vary | 13 | Fluid intelligence (3/3) | 0.1253 | 0.0042 | 0.003* |
| UKB | Vary | 14 | Digits remembered | 0.1466 | 0.0188 | 0.012* |
| UKB | Vary | 15 | Touchscreen duration | 0.1077 | 0.0151 | 0.009* |
| HCP | Fixed | 1 | PicVocab_AgeAdj | 0.005 | 0.005 | 0.615 |
| HCP | Fixed | 2 | ReadEng_Unadj | 0.079 | 0.055 | 0.343 |
| HCP | Fixed | 3 | ReadEng_AgeAdj | 0.005 | 0.006 | 0.598 |
| HCP | Fixed | 4 | PicVocab_Unadj | 0.004 | 0.003 | 0.839 |
| HCP | Fixed | 5 | WM_Task_Acc | 0.070 | 0.036 | 0.184 |
| HCP | Fixed | 6 | PMAT24_A_CR | 0.011 | 0.019 | 0.393 |
| HCP | Fixed | 7 | Relational_Task_Acc | 0.016 | 0.017 | 0.945 |
| HCP | Fixed | 8 | ListSort_Unadj | 0.106 | 0.083 | 0.527 |
| HCP | Fixed | 9 | ListSort_AgeAdj | 0.191 | 0.097 | 0.398 |
| HCP | Fixed | 10 | PicSeq_AgeAdj | 0.015 | 0.012 | 0.577 |
| HCP | Fixed | 11 | PicSeq_Unadj | 0.009 | 0.013 | 0.862 |
| HCP | Fixed | 12 | VSPLOT_TC | 0.009 | 0.007 | 0.615 |
| HCP | Fixed | 13 | VSPLOT_OFF | 0.004 | 0.016 | 0.092 |
| HCP | Fixed | 14 | PMAT24_A_SI | 0.011 | 0.017 | 0.667 |
| HCP | Fixed | 15 | Language_Task_Acc | 0.054 | 0.018 | 0.039* |
| HCP | Vary | 1 | PicVocab_AgeAdj | 0.006 | 0.003 | 0.284 |
| HCP | Vary | 2 | ReadEng_Unadj | 0.118 | 0.078 | 0.422 |
| HCP | Vary | 3 | ReadEng_AgeAdj | 0.076 | 0.05 | 0.615 |
| HCP | Vary | 4 | PicVocab_Unadj | 0.012 | 0.005 | 0.177 |
| HCP | Vary | 5 | WM_Task_Acc | 0.162 | 0.084 | 0.171 |
| HCP | Vary | 6 | PMAT24_A_CR | 0.111 | 0.054 | 0.266 |
| HCP | Vary | 7 | Relational_Task_Acc | 0.030 | 0.004 | 0.012* |
| HCP | Vary | 8 | ListSort_Unadj | 0.403 | 0.103 | 0.108 |
| HCP | Vary | 9 | ListSort_AgeAdj | 0.357 | 0.089 | 0.069 |
| HCP | Vary | 10 | PicSeq_AgeAdj | 0.015 | 0.016 | 0.901 |
| HCP | Vary | 11 | PicSeq_Unadj | 0.103 | 0.056 | 0.500 |
| HCP | Vary | 12 | VSPLOT_TC | 0.014 | 0.007 | 0.284 |
| HCP | Vary | 13 | VSPLOT_OFF | 0.125 | 0.037 | 0.033* |
| HCP | Vary | 14 | PMAT24_A_SI | 0.042 | 0.047 | 0.933 |
| HCP | Vary | 15 | Language_Task_Acc | 0.148 | 0.033 | 0.049* |

**statistically significant results*

## References


Ahrends, C., Woolrich, M., & Vidaurre, D. (2024). Predicting individual traits from models of brain dynamics accurately and reliably using the Fisher kernel. *ELife*. https://doi.org/10.7554/eLife.95125.1

Jaakkola, T., Diekhans, M., & Haussler, D. (2000). A discriminative framework for detecting remote protein homologies. *Journal of Computational Biology*, *7*(1–2), 95–114.

Jaakkola, T., & Haussler, D. (1998). Exploiting generative models in discriminative classifiers. *Advances in Neural Information Processing Systems*, *11*, 487–493. https://doi.org/10.5555/3009055.3009124

Shawe-Taylor, J., & Cristianini, N. (2004). *Kernel methods for pattern analysis*. Cambridge university press.
